# High Intensity Interval Training in a Real World Setting: A Randomized Controlled Feasibility Study in Overweight Inactive Adults, Measuring Change in Maximal Oxygen Uptake

**DOI:** 10.1371/journal.pone.0083256

**Published:** 2014-01-13

**Authors:** Helen Lunt, Nick Draper, Helen C. Marshall, Florence J. Logan, Michael J. Hamlin, Jeremy P. Shearman, James D. Cotter, Nicholas E. Kimber, Gavin Blackwell, Christopher M. A. Frampton

**Affiliations:** 1 Canterbury District Health Board, Christchurch, New Zealand; 2 University of Canterbury, Christchurch, New Zealand; 3 Lincoln University, Lincoln, New Zealand; 4 Christchurch Polytechnic Institute of Technology, Christchurch, New Zealand; 5 University of Otago, Dunedin, New Zealand; 6 University of Otago Christchurch, Christchurch, New Zealand; CUNY, United States of America

## Abstract

**Background:**

In research clinic settings, overweight adults undertaking HIIT (high intensity interval training) improve their fitness as effectively as those undertaking conventional walking programs but can do so within a shorter time spent exercising. We undertook a randomized controlled feasibility (pilot) study aimed at extending HIIT into a real world setting by recruiting overweight/obese, inactive adults into a group based activity program, held in a community park.

**Methods:**

Participants were allocated into one of three groups. The two interventions, aerobic interval training and maximal volitional interval training, were compared with an active control group undertaking walking based exercise. Supervised group sessions (36 per intervention) were held outdoors. Cardiorespiratory fitness was measured using VO_2_max (maximal oxygen uptake, results expressed in ml/min/kg), before and after the 12 week interventions.

**Results:**

On ITT (intention to treat) analyses, baseline (N = 49) and exit (N = 39) 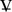
O_2_ was 25.3±4.5 and 25.3±3.9, respectively. Participant allocation and baseline/exit VO_2_max by group was as follows: Aerobic interval training N =  16, 24.2±4.8/25.6±4.8; maximal volitional interval training N = 16, 25.0±2.8/25.2±3.4; walking N = 17, 26.5±5.3/25.2±3.6. The post intervention change in VO_2_max was +1.01 in the aerobic interval training, −0.06 in the maximal volitional interval training and −1.03 in the walking subgroups. The aerobic interval training subgroup increased VO_2_max compared to walking (p = 0.03). The actual (observed, rather than prescribed) time spent exercising (minutes per week, ITT analysis) was 74 for aerobic interval training, 45 for maximal volitional interval training and 116 for walking (p =  0.001). On descriptive analysis, the walking subgroup had the fewest adverse events.

**Conclusions:**

In contrast to earlier studies, the improvement in cardiorespiratory fitness in a cohort of overweight/obese participants undertaking aerobic interval training in a real world setting was modest. The most likely reason for this finding relates to reduced adherence to the exercise program, when moving beyond the research clinic setting.

**Trial Registration:**

ACTR.org.au ACTRN12610000295044

## Introduction

Physical activity has multiple health benefits and is a key component of many lifestyle programs aimed at improving physical fitness and reducing cardio-metabolic risk [1]–[6]. Barriers to achieving a regular physical activity program include lack of time, lack of access to exercise facilities and lack of motivation [7],[8]. The ‘lack of time’ barrier has been addressed recently both in patients with chronic conditions and also in those at risk of cardiometabolic disease by the introduction of high intensity activity programs, which reduce the time required to exercise compared to low intensity programs [9],[10]. A common model for studies using HIIT (high intensity interval training) in populations with or at risk of cardiac disease is an AIT (aerobic interval training) prescription of 4 or 5 work bouts each of 3 to 4 minutes duration, done at around 85% to 90% of maximal heart rate, followed by a recovery period. In addition to AIT another form of HIIT, maximal volitional interval training (MVIT) has also been well described. MVIT, or low volume high intensity training, involves repetitions of ‘all out’ maximal volitional effort, typically for 30 seconds, followed by a recovery period. The optimal prescription for the recovery periods following both of these high intensity activities is unknown, both in terms of time interval and also exercise intensity, however a bout of high intensity activity is typically followed by 3 to 4 minutes recovery at around 60% to 70% maximum HR (heart rate).

Two preliminary HIIT studies in inactive adult participants, one laboratory based and one in young males, measured change in maximum oxygen uptake (VO_2_max), an objective and reproducible measure of cardiorespiratory fitness (CRF) [11], and a predictor of morbidity and mortality [12],[13], as their primary outcome [14],[15]. Results from both studies were positive. We chose to base our AIT exercise intervention on the study by Tjonna [14], which used 4 minute intervals at 90% maximal HR, followed by three minutes of active recovery at 70% maximal HR.

Although multiple studies on selected populations in controlled settings have shown HIIT to be an effective method of improving markers of cardiometabolic fitness [10],[16], HIIT has yet to be applied to a real world community setting using moderately unselected participants. The purpose of the current study was to extend and translate the HIIT concept into a real world community setting, by undertaking a randomized controlled trial (RCT) feasibility study. In keeping with the real world objectives of the study, participants were selected because they were at high risk of cardio-metabolic disease and the study environment was specifically chosen to be low cost. Also a clinically meaningful primary end point, change in VO_2_max, was measured at entry into and exit from this 12 week study.

The hypothesis of this pragmatic study was that interval training using either a) four minute repetitions of aerobic interval training aiming to get to 85 to 95% of maximal heart rate, or b) maximal volitional exercise using 30 seconds of ‘all out’ exercise, could reduce the amount of exercise time required to achieve an improvement in VO_2_max when compared to steady walking, in previously inactive, overweight and obese adults exercising in a community park setting.

## Methods

The protocol for this trial and supporting CONSORT checklist are available as supporting information; see Checklist S1 and Protocol S1.

### Participants

**Figure 1:**
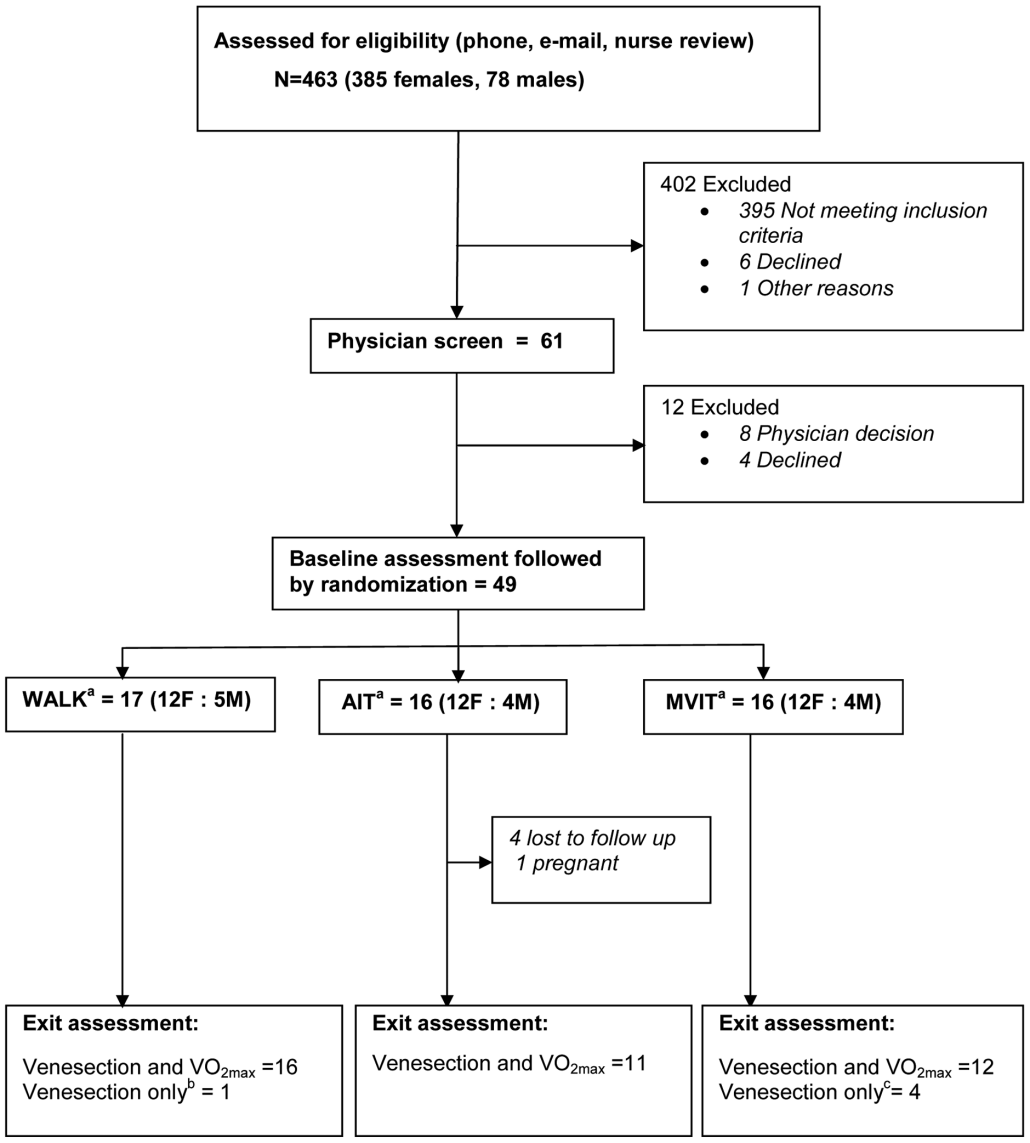
Flow of participants through each stage of the trial. ^a^ WALK  =  walking to 65–75% of heart rate max, AIT  =  aerobic interval training to a 85–95% of heart rate max MVIT  =  Maximal volitional intensity training (‘all out’ exercise). ^b^ Participant unable to undertake exit VO_2_max because of injury unrelated to the study. ^c^ Four participants did not undertake exit VO_2_max: Exercise related injury n = 1, chest infection n = 2, participant exited early as dissatisfied with exercise program n = 1.

Recruitment was undertaken through advertising in the community and also at the researchers' host institutions. Inclusion criteria were assessed during the screening visit. Criteria were; age 35–60 years, BMI (body mass index) 28–40 kg/m^2^, no regular activity (defined as achieving less than 2x30 minutes of moderate intensity physical activity each week), no plans for a change in lifestyle outside the study's group exercise sessions, no barriers to either participation in group physical activity sessions or to undertaking treadmill based VO_2_max assessments and no major health problems that might preclude safe participation in the study. Initial screening was undertaken by a team of research nurses, followed by physician assessment (see Figure 1. The Upper South B Regional Ethics Committee, New Zealand, approved this study (URB/09/12/069). All participants gave written informed consent.

### Physical activity program

After baseline assessment, participants were randomized 1∶1∶1 to one of three parallel exercise groups: A group undertaking walking based low intensity exercise training i.e. an ‘active control group’ undertaking a standard exercise intervention (WALK); a group undertaking aerobic interval training (AIT); a group undertaking maximal volitional intensity training (MVIT). The AIT exercise prescription was designed to be isocaloric when compared to WALK and was based on a protocol previously shown to improve VO_2_max in heart failure patients over a 12 week period [17]. The MVIT program was based on the protocol developed by Burgomaster [18], which used a lower calorie expenditure than either WALK or AIT. The original six week Burgomaster protocol was modified for the current study to take into account its longer (12 week) duration. Further detail about the three exercise prescriptions is discussed below.

There were three scheduled group exercise sessions per week over the twelve weeks of study, giving a total of 36 scheduled sessions. All three exercise prescriptions were designed to maintain a progressive overload over the twelve weeks of study. WALK and AIT were HR (heart rate) based and submaximal, thus as participants improved their fitness they had to work harder to maintain their HR within the required HR zone. For the MVIT group, progression was provided by an increase in the number of repetitions and duration of each repetition though the course of the study. The time duration of each MVIT repetition was however too short to allow meaningful HR monitoring in this subgroup. WALK was a walking based prescription of a 33 minute walk which aimed to achieve a HR of 65–75% HR_max_ (maximum heart rate). The AIT group undertook 4 minutes high intensity exercise, either fast walking or jogging depending on fitness, followed by 3 minutes at walking pace over four repetitions. The aim of the 4 minute high intensity repetitions was to achieve a HR of 85–95% HR_max_ and this typically required a period of 10–20 seconds at the start of the 4 minute repetition, before the HR rose to the target zone. The MVIT group's exercise prescription was designed to progress, so that exercise overload was maintained during the course of the 12 week study. Participants started with 30 second repetitions of volitional maximal intensity walking or jogging up a slope, followed by a recovery period of four minutes walking. Initially this repetition was undertaken three times but participants were encouraged to increase the duration and number of repetitions over the 12 week study, aiming for up to six repetitions of 45 seconds of maximal volitional activity.

All participants completed a 10 minute warm up and 5 minute cool down at every supervised exercise session. When warm up and cool down periods were included in the exercise prescription, the WALK group prescription was therefore for 48 minutes of exercise per session, which is consistent with the recommendation that adults exercise for at least 150 minutes per week [3]. The AIT group prescription was for 40 minutes. The duration of the MVIT sessions was designed to increase gradually over twelve weeks, starting at 24.5 minutes per session and aiming for a maximum duration per session of up to 40 minutes in the final week.

**Figure 2:**
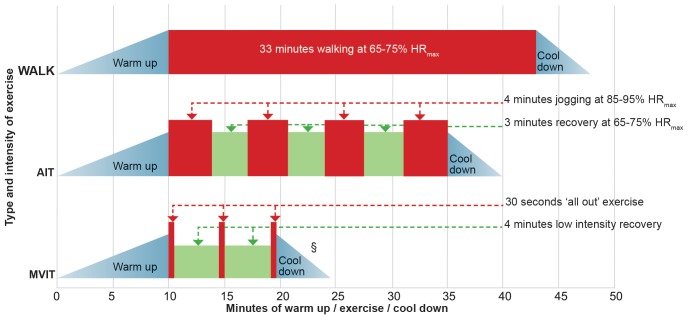
Schematic representation of the three exercise prescriptions allocated 1:1∶1 at randomisation. ^§^ Number of repetitions in MVIT group increased over twelve weeks, as participant's fitness levels improved. WALK  =  walking, AIT  =  aerobic interval training, MVIT  =  maximal volitional intensity training.

In summary exercise time per session (minutes) was allocated as follows: WALK [10 warm up + 33 moderate walk + 5 cool down] 48 minutes; AIT [10 warm up + 4 high intensity +3 recovery +4 high intensity +3 recovery + 4 high intensity +3 recovery + 4 high intensity + 5 cool down] 40 minutes; MVIT (beginning sessions) [10 warm up + 0.5 ‘all out’ exercise + 4 recovery + 0.5 ‘all out’ exercise + 4 recovery + 0.5 ‘all out’ exercise + 5 recovery/cool down] 24.5 minutes. The exercise prescriptions are also summarized in Figure 2.

HR monitors (Polar FSI, FS3c, Edge; Finland) were provided to WALK and AIT participants for the duration of the exercise prescription, allowing them to exercise within their individualized HR zones. Prior to study onset, it was determined that the exercise prescription would be reviewed and potentially modified if two or more participants in an exercise group developed injuries related to exercise intensity or duration. Further detail about the monitoring of adverse events/harms is given in Text S1.

Exercise sessions were held outdoors in a 459 acre public park (Hagley Park, Christchurch) in the evening, in Autumn/Winter, irrespective of weather conditions. The park is predominantly flat and grassed but has a small artificial slope with a 15% incline, used for some MVIT group activities. Two experienced applied exercise physiology students (‘exercise group leaders’) led each exercise session for each group. These six exercise group leaders were in turn trained and supervised by two senior exercise physiologists (HM and ND). The exercise group leaders supervised only their allocated group of participants throughout the study. If participants were unable to attend any of their 36 scheduled group sessions, they were offered ‘catch up’ sessions at the public park with their allocated group, once a week. If unable to attend this, participants were then asked to undertake individual unsupervised exercise sessions, at a time and venue convenient to the participant. Details of methodology used to document adherence with the exercise prescription are given in Text S1.

### Outcome measures

Measurements were undertaken at baseline and at the exit visit. Participants attended the research clinic after a minimum of an eight hour overnight fast, for clinical and anthropometric measurements related to the secondary outcome measures; change in BMI, waist circumference, blood pressure, insulin sensitivity (%S) and fasting lipids (total cholesterol, HDL-cholesterol and triglycerides). Venous samples were taken from the antecubital fossa, spun on site in a refrigerated centrifuge then plasma stored at minus 20° centigrade for later batched analysis at an accredited laboratory. These secondary measures and further ancillary measures such as the SF36 quality of life questionnaire [19], food diary and incidental physical activity measured using a pedometer, were assessed to ensure that any apparent health benefits associated with improvement in VO_2_max were not counteracted by changes in related parameters. Hemoglobin and hematocrit was also assessed, in part to exclude any participant with overt anemia and in part because of the impact of changes in hematocrit on VO_2_max. Further detail about the measurement of the above variables is given in Text S1.

The primary outcome, VO_2_max, was measured in the exercise laboratory, typically on the same day as the research clinic visit for the abovementioned biochemical and anthropometric measures. VO_2_max was assessed using a maximal treadmill test based on the modified Bruce protocol [20], with online gas analysis (Metalyser, Cortex, Biophysik, Leipzig, Germany). The analyzer was turned on for at least 45 minutes before calibration and testing. Pressure was calibrated via electronic barometer (GPB300 Greisinder electronic Germany) and flow volume was calibrated via Hans Rudolph 3 Litre calibrated syringe. Gas calibration was done using a two step calibration process; room air at 20.93% and 0.03% CO_2_ and alpha standard calibrated gas bottle with concentrations of 15.80%±0.08 O_2_ and 4.98%±0.02 C0_2_ (BOC New Zealand). Criteria for achieving VO_2_max were at least two of the following: A leveling off of oxygen uptake, a respiratory exchange ratio >1.15 and a HR within 10 beats per minute of age-predicted maximum.

All measurements were undertaken by trained researchers using calibrated equipment. Details of other measurement methodologies, including secondary and ancillary measurements such as measurement of incidental activity, measurements related to study adherence and also monitoring of adverse events/harms, are given in Text S1.

### Blinding, including allocation concealment

There were separate intervention and assessment teams, as described in Table 1, which also summarizes blinding procedures and allocation concealment. Further detail about blinding of clinical assessors is given in Text S1. Participants were booked for their baseline VO_2_max measurement by an on-site exercise physiologist (HM), who was blind to the block randomization process. Raw exercise treadmill data were transferred electronically for interpretation by a single off site exercise physiologist (JC), who was responsible for determining all study VO_2_max values. This exercise physiologist then relayed VO_2_max results to the off-site biostatistician (CF), who randomly assigned the participant to one of the three exercise groups using baseline VO_2_max for stratification. Assignment was notified to the supervising exercise physiologists 24 hours prior to the first booked exercise session. Participants were notified of their group allocation at their first exercise visit but were not informed about how their group allocation translated into an exercise prescription until after they had separated into their individual exercise groups. Participants were repeatedly reminded at their visits, not to disclose their group assignment to assessment team members. To enhance blinding of the primary endpoint, individual VO_2_max results were not made available to participating individuals or to the assessing or interventional research teams, until database lock had been achieved.

**Table 1:** Description of researchers' primary role in trial conduct and mechanisms for blinding.

Description of researcher(s)	1° role in trial conduct	Mechanism(s) to ensure blinding
Exercise physiologists supervising exercise interventions, including supervision of exercise group leaders (ND and HM)	Administer and monitor exercise interventions and measure adherence to prescribed exercise, including heart rate response to exercise	Following participant randomization, researchers excluded from data collection, other than recording exercise attendance and heart rate. Downloaded heart rate data not analyzed until intervention completed.
Exercise physiologists undertaking VO_2_max tests (GB, MH, JS, NK)	Responsible for VO_2_max testing at exercise laboratory and collection of raw data	Blind to group allocation
Exercise physiologist responsible for calculation of VO_2_max i.e. primary endpoint (JC)	VO_2_max calculation using raw data forwarded electronically from the exercise laboratory	Blind to participant identity and to group allocation and working at a remote site. Calculated VO_2_max results forwarded electronically to biostatistician at baseline and at follow up. Following calculation of baseline VO_2_max, researcher had no further access to these baseline data
Physician (HL)	Documentation of harms/adverse events	Blind to group allocation. Harms documentation locked with biostatistician after completion of exit interview
Research nurses (led by FL)	Collection of laboratory and pedometer data (used to measure non prescribed incidental activity)	Blind to group allocation
Dietitian	Interview participants about food records, analyze macronutrient intake	Blind to group allocation

### Statistical methods, including analyses

Randomization into one of the three groups, WALK, AIT or MVIT, was stratified by gender and by VO_2_max above or below age-gender 20^th^ centiles [21]. This study was designed as a non-inferiority study whereby if either MVIT or AIT could be shown to be non-inferior with respect to VO_2_max improvements over 12 weeks then the advantages they proffer in terms of reduced exercise time would indicate they would be attractive potential options for physical activity programs. Assuming a standard deviation of the change in VO_2_max of approximately 3 ml/kg/min [14], a sample size of 16 in each group would enable non-inferiority to be concluded if the reduction in VO_2_max improvement was not more than 3 ml/kg/min, (two tailed α = 0.05, 80% power). Two sided 95% confidence intervals are used to summarize the differences in the changes between MVIT and AIT, and WALK from which non-inferiority in terms of the primary outcome VO_2_max can be determined [22]. These confidence intervals were generated from a general linear model which included the baseline VO_2_max and randomization stratum as covariates and was based on the per-protocol (PP) population with confirmatory analysis undertaken on the intention-to-treat (ITT) population. All other outcome measures are analyzed using standard statistical hypothesis testing for superiority, using the same general linear model as for the primary outcome, with the relevant baseline assessment and stratum as covariates with a two-sided P-value <0.05 taken to indicate statistical significance. Analyses of these other outcome measures were undertaken using an ITT approach. Participants were pre-defined as following the intervention i.e. PP, if they remained in active contact with the exercise team and undertook the final treadmill testing and, if unable to attend group exercise sessions in any given week, undertook unsupervised exercise sessions in their own time so that in total they completed more than 70% of their prescribed program. All statistical analyses were undertaken using SPSS V19.

Text S1 provides further methodological details about clinical and anthropometric measurements and questionnaires, adherence with the exercise prescription, adherence with the request not to undertake lifestyle changes outside the interventions sessions, monitoring of adverse events/harms and blinding of clinical assessors to details of the exercise prescription.

## Results

Participants were recruited between February and April 2010 and the exercise intervention occurred between April and July 2010. Participant flow through the recruitment, assessment and intervention phases is shown in Figure 1. The female: male response rate to the advertising campaign was high as was the attrition rate between initial response to advertisement and physician screen (Figure 1). The reasons for not meeting inclusion criteria by gender (female: male) were as follows; unable to attend group activities at pre-specified times (125∶23), health problems (58∶14), outside BMI range (64∶6), research team unable to contact (43∶12), and not physically inactive (24∶2). Six potential participants elected to discontinue screening when study details were described. One advertisement responder, identified as a researcher's relative, was excluded. A further 12 potential participants were excluded after physician assessment because of health reasons (N = 6), the presence of benign cardiac arrhythmias that might impede monitoring of heart rate (N = 2), and participant's choice (N = 4).

There were 49 participants aged 35 to 59 years, 90% of whom were of New Zealand European ethnicity. The baseline BMI range was 27.7–39.3 kg/m^2^. The mean baseline 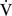
VO2max was 25.3±4.5 ml/min/kg. Of the 36 females, 29 had a VO_2_max below the age-gender 20^th^ centile and of the 13 males, 11 were below their age-gender 20^th^ centile. These baseline results confirm that participants had low cardiorespiratory fitness and were overweight or obese. There were no current smokers, 19 (39%) were ex-smokers and smoking status remained unchanged during the study. Regarding medications, 10 participants were receiving antidepressants and 7 were taking cardiovascular risk reduction medications. Two participants had Type 2 diabetes. No participant required a change in prescribed medications during the study. The 49 participants were randomized as follows; 17 into WALK and 16 into each of the MVIT and AIT groups. Baseline clinical characteristics were well-matched across groups (Table 2). The days of the week on which the group exercise sessions were held were determined after randomization by participant consensus. They were Monday, Wednesday and Friday evenings for the AIT group and Monday, Tuesday and Friday evenings for the WALK and MVIT groups.

**Table 2:** Baseline characteristics for the entire cohort and the subgroup completing the exercise protocol.

	WALK^a^		AIT^a^		MVIT^a^	
Characteristics *(SD when appropriate)*	ITT^b^ (n = 17)	PP^b^ (n = 14)	ITT^b^ (n = 16)	PP^b^ (n = 9)	ITT^b^ (n = 16)	PP^b^ (n = 9)
Mean age (years)	46.3 *(5.4)*	45.7 *(5.7)*	48.2 *(5.6)*	49.1*(4.8)*	50.3*(8.0)*	53.5*(7.2)*
Gender (M:F)	5∶12	9∶5	4∶12	6∶3	4∶12	6∶3
Mean VO_2_max (ml/kg/min)	26.5*(5.3)*	27.1*(5.6)*	24.2*(4.8)*	24.7 *(6.1)*	25.0*(2.8)*	25.1 *(2.7)*
Mean BMI (kg/m^2^)	32.7*(3.4)*	32.9 *(3.7)*	32.1*(3.1)*	32.4 *(3.0)*	32.4*(2.9)*	32.2 *(3.7)*
Mean systolic/diastolic blood pressure (mm Hg)	133.0/84.7 *(20.2/8.6)*	127.9/83.7 (*13.7/8.2)*	118.7/77.4 *(13.7/8.0)*	121.6/80.1 *(10.4/6.3)*	129.4/80.6 *(13.8/10.9)*	124.8/76.6 *(12.9/9.5)*
On medications (n - %)	11 (65%)	8 (57%)	9 (56%)	6 (67%)	10 (63%)	5 (56%)
On multiple medications (n - %)	4 (25%)	4 (29%)	2 (13%)	1 (13%)	5 (31%)	2 (22%)
Number (%) with +ve PAR-Q screen^c^	7 (41%)	7 (50%)	3 (19%)	3 (33%)	4 (25%)	2 (22%)

^a^ WALK  =  Low intensity walking, AIT  =  Aerobic interval training, MVIT  =  Maximal volitional intensity training.

^b^ ITT (intention to treat) includes all 49 randomized participants, PP (per protocol) includes the 32 participants who completed >70% of their exercise prescription (see text for more detail).

^c^ A positive response indicates possible health problems, with the recommendation that a medical screen is undertaken prior to increased physical activity.

At week 10, three participants in the MVIT group had developed injuries considered to be related to the intensity of the intervention. According to predefined criteria the supervising exercise physiologists and physician decided not to make the planned increase in exercise duration and load for this group, during weeks 11 and 12. Up until this time point in the study, the MVIT protocol appeared to work well for participants.

Ten participants did not complete the study and did not return for exit evaluations and a further 7 completed less than 70% of the physical activity program. Consequently, the PP analysis included 32 participants; 14, 9 and 9 participants from the WALK, AIT and MVIT groups respectively. Baseline and exit results for VO_2_max and secondary outcomes for the entire cohort are shown in Table 3. Baseline characteristics suggest that many participants had features of the metabolic syndrome. Table 4 shows the same baseline and exit results by allocated group. Regarding the primary outcome, the AIT and MVIT groups showed non-inferiority to the WALK group on both PP and ITT analyses of the VO_2_max changes and hence were examined for possible superiority using both PP (n = 32) and ITT (n = 49) analyses (Table 5). With ITT analysis, the AIT group but not the MVIT group improved their VO_2_max more than the WALK group (p = 0.027) whereas the difference between the MVIT and WALK groups was not statistically significant for either analysis (Table 5). On review of raw data, the different levels of significance between the ITT and PP analyses related primarily to three participants in the ITT analysis who showed large changes in VO_2_max but were not eligible for inclusion in the PP analysis: One WALK participant dropped their VO_2_max by 3.89 ml/min/kg and in the AIT group there were rises in VO_2_max of 2.73 ml/min/kg and 3.77 ml/min/kg, respectively. One participant in the AIT group did not achieve plateau VO_2_max on the exit test but met the two other pre-defined criteria for VO_2_max (HR and respiratory exchange ratio).

**Table 3:** Primary and secondary outcomes and other relevant parameters before and after the 12 week exercise program for the entire cohort.

	Total Group ITT N = 49			Total Group PP N = 32		
	Baseline	Exit	*P-value*	Baseline	Exit	*P-value*
**Primary outcome**						
VO_2_max (ml/min/kg)	25.3(4.5)	25.3(3.9)	*0.825*	25.9(5.1)	26.0(4.3)	*0.863*
**Secondary outcomes**						
Peak heart rate – treadmill (bpm)	174.9(9.6)	172.6(9.2)	*0.004*	174.6(11.0)	171.8(10.2)	*0.011*
BMI kg/m^2^	32.4(3.1)	32.3(3.0)	*0.302*	32.6(3.4)	32.4(3.4)	*0.218*
Waist circumference (cm)	103.3(11.1)	100.3(11.0)	*<0.001*	105.6(11.1)	101.6(11.6)	*<0.001*
% body fat	39.8(5.0)	38.9(5.3)	*<0.001*	38.7(5.3)	37.8(5.5)	*<0.001*
Systolic blood pressure (mmHg)	127.1(17.1)	122.3(15.9)	*0.001*	125.3(12.6)	120.1(11.3)	*0.003*
Diastolic blood pressure (mmHg)	81.0(9.5)	77.4(10.4)	*0.004*	80.7(8.4)	76.0(7.7)	*<0.001*
%S HOMA	76.1(45.8)	81.5(42.6)	*0.174*	72.9(46.2)	79.2(39.1)	*0.207*
Total cholesterol (mg/dL)	216.0(45.1)	208.1(40.9)	*0.025*	212.4(38.3)	200(36.5)	*0.012*
Triglycerides (mg/dL)	68.2(39.6)	68.4(32.7)	*0.968*	73.2(42.9)	66.6(28.2)	*0.171*
HDL cholesterol (mg/dL)	49.9(11.4)	48.3(12.1)	*0.040*	47.4(11.0)	46.5(11.0)	*0.237*
**Additional measures**						
Energy intake (kJ)	9177(2403)	8500(2817)	*0.040*	9760(2580)	9149(3093)	*0.186*
SF 36 – mental health^a^	73.8(13.5)	75.4(13.8)	*0.307*	73.8(13.5)	75.4(13.8)	*0.076*
SF 36 – physical health^a^	75.4(11.1)	73.4(15.0)	*0.313*	75.4(11.1)	73.4(15.0)	*0.082*
SF 36 – body pain^a^	80.1(15.7)	69.0(21.2)	*0.003*	77.5(14.8)	71.5(18.2)	*0.161*

Values are means (SD). Intention to treat population N = 49; WALK n = 17, AIT n = 16, MVIT n = 16. Per-protocol population N = 32; WALK n = 14, AIT n = 9, MVIT n = 9.

^a^ An increase in SF36 score indicates improved quality of life.

**Table 4:** Primary and secondary outcomes and other relevant parameters before and after the 12 week exercise program, by group.

	WALK		AIT		MVIT	
	Baseline	Exit	Baseline	Exit	Baseline	Exit
**Primary outcome**						
VO_2_max (ml/min/kg)^a^	27.1(5.6)	25.8(3.7)	24.7(6.1)	26.5(5.8)	25.1(2.7)	25.7(3.6)
VO_2_max (ml/min/kg)^b^	26.5(5.3)	25.2(3.6)	24.2(4.8)	25.6(4.8)	25.0(2.8)	25.2(3.4)
**Secondary outcomes**						
Peak heart rate – treadmill (bpm)	179.0(8.9)	176.9(8.7)	174.0(9.9)	172.8(10.5)	171.5(9.0)	168.0(6.2)
BMI kg/m^2^	32.7(3.4)	32.6(3.4)	32.1(3.1)	32.1(3.0)	32.4(2.9)	32.3(2.9)
Waist circumference (cm)	105.9(11.2)	102.3(10.7)	100.4(11.7)	98.6(12.5)	103.5(10.4)	100.0(9.9)
% body fat	39.5(5.2)	39.0(5.3)	39.5(5.4)	39.0(5.6)	40.2(4.7)	39.1(5.2)
Systolic blood pressure (mmHg)	133.0(20.1)	127.8(21.3)	118.7(13.7)	115.8(11.8)	129.4(13.8)	122.9(10.4)
Diastolic blood pressure (mmHg)	84.7(8.6)	82.8(13.6)	77.4(8.0)	74.0(6.8)	80.6(10.9)	75.2(7.3)
%S HOMA	65.5(30.1)	71.8(37.0)	89.5(59.0)	88.2(47.4)	74.0(44.1)	75.2(44.0)
Total cholesterol (mg/dL)	207(33)	191(37)	210(42)	209(31)	231(57)	224(49)
Triglycerides (mg/dL)	69(32)	63(21)	67(56)	66(37)	69(28)	77(38)
HDL cholesterol (mg/dL)	48(9)	46(9)	51(11)	50(12)	51(14)	49(15)
**Additional measures**						
Energy intake (kJ)	10222(2503)	8380(2409)	8463(2616)	8345(2508)	8706(1693)	8717(3498)
SF 36 – mental health^c^	75.6(14.8)	76.5(11.3)	71.9(14.7)	72.4(15.6)	73.8(11.0)	77.3(14.7)
SF 36 – physical health^c^	76.6(10.3)	72.0(18.6)	74.9(13.3)	75.1(11.9)	74.8(10.3)	73.3(14.2)
SF 36 – body pain^c^	75.6(16.1)	70.1(25.0)	85.0(14.0)	75.5(15.1)	80.1(16.3)	61.4(20.8)

Values are means (SD).

^a^ Per-protocol population WALK n = 14, AIT n = 9, MVIT n = 9.

^b^ Intention to treat population WALK n = 17, AIT n = 16, MVIT n = 16.

^c^ An increase in SF36 score indicates improved quality of life.

**Table 5:** Changes (post minus pre) in primary and secondary outcome measures and comparison between low intensity exercise and high intensity interval and maximal volitional intensity training.

	WALK^a^	AIT^a^	Diff 95% CI	P	MVIT^a^	Diff 95% CI	P
**Primary outcome**			AIT - WALK			MVIT - WALK	
VO_2_max (ml/min/kg)^b^	−0.87	1.40	2.27(−0.44–4.97)	0.096	0.25	1.12(−1.39–3.63)	0.37
VO_2_max (ml/min/kg)^c^	−1.03	1.01	2.03(0.25–3.82)	0.027	−0.06	0.97(−0.76–2.70)	0.27
**Secondary outcomes** c							
Peak heart rate – treadmill (bpm)	−1.12	−1.24	−0.11(−3.72–3.49)	0.95	−3.78	−2.67(−6.42–1.08)	0.16
BMI (kg/m^2^)	−0.09	−0.02	0.06(−0.29–0.42)	0.72	0.00	0.09(−0.27–0.44)	0.62
Waist circumference (cm)	−4.03	−2.46	1.57(−1.22–4.36)	0.26	−4.27	−0.23(−2.95–2.48)	0.86
Body fat (%)	−0.80	−0.71	0.10(−0.70–0.89)	0.81	−1.27	−0.46(−1.26–0.33)	0.25
Systolic blood pressure (mmHg)	−3.90	−4.64	−0.77(−7.64–6.11)	0.82	−5.84	−1.97(−8.48–4.53)	0.54
Diastolic blood pressure (mmHg)	−2.33	−5.61	−3.28(−8.91–2.34)	0.25	−6.94	−4.61(−10.05–0.84)	0.10
S HOMA (%)	4.33	3.39	−0.94(−19.5–17.6)	0.92	11.94	7.6(−10.7–25.9)	0.41
Total cholesterol (mg/dL)	−13.4	1.4	14.9(0.4–29.3)	0.045	2.2	15.6(0.8–30.5)	0.040
Triglycerides (mg/dL)	−2.2	1.4	3.6(−12.4–19.6)	0.65	13.2	15.4(−0.7–31.5)	0.061
HDL cholesterol (mg/dL)	−1.8	−0.7	1.1(−2.6–4.8)	0.55	−2.9	−1.1(−4.8–2.6)	0.56
**Additional measures**							
Energy intake (kJ)	−2233	−528	1706(153–3259)	0.032	−520	1713(179–3248)	0.030
SF 36 – mental health	1.3	0.5	−0.8(−7.9–6.3)	0.82	3.6	2.3(−4.8–9.4)	0.52
SF 36 – physical health	−1.3	3.6	4.9(−4.5–14.3)	0.30	2.2	3.6(−5.9–13.0)	0.45
SF 36 – body pain	−8.8	−4.6	4.1(−11.6–19.5)	0.60	−17.7	−8.9(−24.3–6.5)	0.25

Values are means (SD).

^a^ WALK  =  low intensity walking, AIT  =  aerobic interval training, MVIT  =  maximal volitional intensity training.

^b^ Per protocol population WALK n = 14, AIT n = 9, MVIT n = 9.

^c^ Intention to treat population WALK n = 17, AIT n = 16, MVIT n = 16.

In order to determine whether there was an increase in aerobic fitness that was independent of the small change in body mass seen over the twelve weeks of study, VO_2_max was also calculated as L/min, rather than as the conventional ml/min/kg, as used in Tables 2 to 5. When considering the group as a whole, there continued to be no significant overall change in VO_2_max, either on ITT (N = 39) or PP (N = 32) analyses. However, when considering the change in AIT VO_2_max (L/min) relative to WALK VO_2_max (L/min) (see also the subgroup comparisons used in Table 5), there was a significant difference favouring AIT on both ITT analysis (p = 0.01) and also on PP analysis (p = 0.04). Comparison of MVIT with WALK continued to show non-significant changes.

As discussed above, Table 3 compares secondary outcome measures at baseline and also exit for all participants, using both ITT and PP analyses. Although there was no significant change in body mass and only a small non-significant fall in self-reported calorie intake, waist circumference fell by 3–4 cm and total body fat fell by an amount equivalent to a 1.0 kg loss in fat mass. Additional beneficial effects of the exercise program included a fall in total cholesterol and blood pressure. The fall in blood pressure may however have been partly related to a reverse ‘Hawthorne effect’, whereby possible initial anxiety about the study was associated with a blood pressure increase at entry into study, which subsequently settled and was not present at the time of exit examination.

The main reason for measuring and comparing secondary outcomes by group (Tables 4 and 5) was to determine whether any apparent benefit of an intervention that seemed to improve VO_2_max relative to the WALK group, might be offset by negative health changes. Apart from the observation that the WALK group's total cholesterol improved more than that of the AIT and MVIT groups, no negative consequences of AIT (or MVIT) were observed. These differences in total cholesterol should be interpreted in the context of a small change in recorded energy intake between WALK and the other two groups, with WALK showing a larger decline over the study period. There are two additional measures not included in the Tables, hematocrit and total SF 36 score. Neither measure changed following the interventions (hematocrit mean pre = 0.43 and mean post = 0.42). Median time interval between last exercise session and exit VO_2_max assessment at the exercise laboratory was one day. Median time interval between last exercise session and fasting data collection at the research clinic was 3 days. There were no statistically significant between group differences in these time intervals.

**Figure 3:**
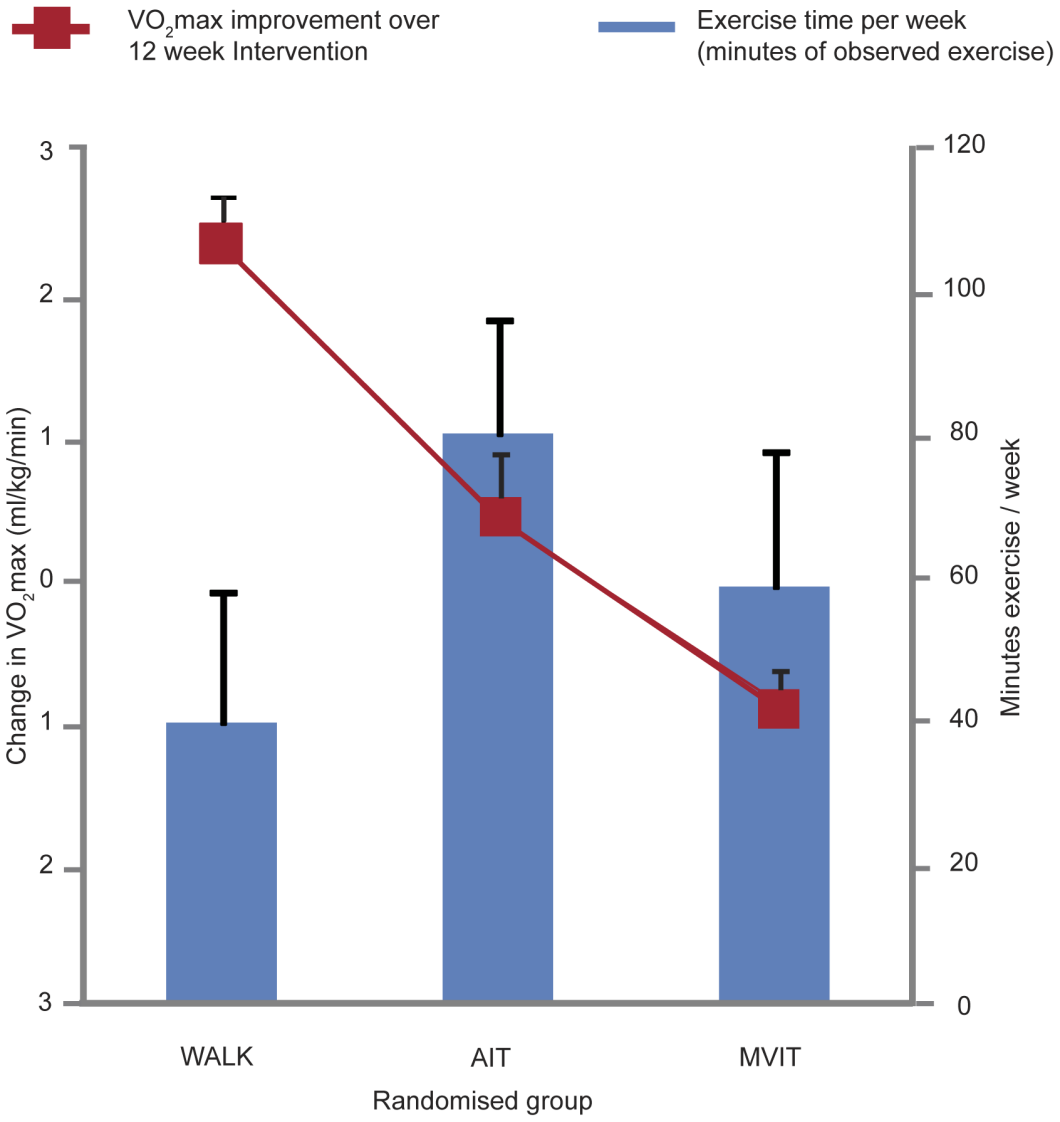
Changes in V0_2_ max and time per week exercising, by treatment group. Intention-to-treat analysis thus data on exercise time includes data from ‘non-adheres’, unable or unwilling to complete the exercise prescription. Data are means and standard errors estimated from the general linear model.

The time spent exercising per session was fixed for the WALK and AIT groups but the session time increased during the 12 week study for the MVIT group as fitness improved (see Methodology section). Although there was only a small difference in the time duration of prescribed exercise between the AIT and WALK groups at 120 and 144 minutes a week respectively (see Table 6), the high attrition rate in the AIT group compared to the WALK group meant that the actual average exercise time by group, when considering data on all 49 participants (i.e. on ITT analysis), differed considerably as shown in Table 6. Thus over the duration of the study the AIT and MVIT groups spent on average 36% less time (74 minutes/week) and 61% less time (45 minutes per week) exercising than the WALK group (116 minutes/week); p<0.001 for both comparisons. Figure 3 compares change in VO_2_max with time spent exercising per week, over the 12 weeks of study. Results from the downloadable HR monitors showed that target HR was achieved by WALK participants 87.7% of the time and AIT participants 73.4% of the time. The maximum achieved heart rate during the MVIT sessions averaged 88.6% of HR_max_ at week 6 and 85.1% of HR_max_ at week 11. The amount of incidental exercise being undertaken outside the formal sessions during the 12 weeks of the study, as measured by pedometer steps, was not significantly different between the groups (WALK, weeks 5 to 6 = 5364, weeks 11 to 12 = 4739; AIT weeks 5 to 6 = 5021, weeks 11 to 12 = 4890; MVIT weeks 5 to 6 = 5693, weeks 11 to 12 = 4969). This relatively low average number of pedometer steps recorded in between the supervised exercise sessions was consistent with recruitment of inactive participants [23]. Adverse events including exercise related injuries are summarized in Table S1 The pre-determined blinding processes incorporated into study design (Table 1), were successfully maintained throughout the study.

**Table 6:** Attendance at exercise sessions and time spent exercising over 12 weeks, for all randomized participants (n = 49).

Exercise group allocation			
	Low intensity walking (WALK)	Aerobic interval training (AIT)	Maximal volitional intensity training (MVIT)
% of sessions attended	75%	59%	75%
Theoretical amount of time (min/week) allocated to exercise sessions over the 12 week study^a^	144	120	90^b^
Actual amount of time (min/week; mean, range) spent exercising over the12 week study^a^	116 (40–144)	74 (7–117)	45 (0–62)
Actual amount of time spent exercising (min/week) during final week of study^a^	80	46	31

^a^ Includes the 10 minute warm up and 5 minute cool down undertaken by all three groups, but excludes time spent exercising outside scheduled exercise groups.

^b^ MVIT participants progressively increased repetitions during the 12 weeks of training. This value is therefore an estimate only.

## Discussion

This study demonstrates that the HIIT concept can be applied in a real world setting (community recruitment, exercise sessions undertaken in a community park). This study therefore extends the positive findings from previous efficacy studies of HIIT in participants at risk of cardiometabolic disease, which have typically taken place in either an exercise facility and/or using individually supervised exercise sessions [14], [15]. The study also demonstrated that it was possible to maintain rigorous blinding procedures whilst undertaking an exercise study in a community setting. An ancillary analysis showed that, when considering the group as a whole, body composition, total cholesterol and blood pressure improved. The magnitude of anthropomorphic change seen in the current study was similar to that seen in another recent high intensity intermittent exercise study, also of twelve weeks duration [24].

The cardiorespiratory benefits seen in this real world study were however modest, with the largest change in VO_2_max, seen in the AIT group on ITT analysis, being equivalent to around a 10% increase in comparative fitness on age/gender centile charts. There are multiple possible reasons for the small observed change in VO_2_max, both between and within groups. Firstly, although overall adherence to the exercise program was in keeping with similar exercise intervention studies [25], it was low in both the AIT and MVIT groups, even in those participants able and willing to undertake an exit VO_2_max assessment (see Table 6). This low level of adherence will impact on group change in VO_2_max. Secondly, participant numbers were small, thus the confidence interval around group differences in VO_2_max was wide (see Table 5). Thirdly, one inclusion criterion was a history of inactivity. Physical inactivity has genetic determinants [26]. The recruitment strategy may have biased recruitment towards inclusion of exercise non responders [27], as some participants may have been inactive because previous attempts at exercise failed to produce positive reinforcement through a feeling of increased fitness. Fourthly, although a twelve week intervention has been reported to be sufficiently long to observe changes in VO_2_max [28], [29], a longer duration study may have shown further improvements in VO_2_max [29]. Fifthly, the maximal pulse rate achieved on VO_2_max testing was less at exit than at baseline. Although participants fulfilled the standard criteria for achieving VO_2_max both at baseline and at exit (see Methodology section above), there may have been subtle differences in participant's maximal exertion between these two measures, that resulted in a slight attenuation of the measured exit VO_2_max values. The maximal achieved heart rate for the 39 participants who undertook the exit VO_2_max was 172 beats per minute (see Table 4), which was nevertheless consistent with the age-predicted average maximum heart rate. There were however no between group differences in maximal achieved pulse rate during the exit VO_2_max, which suggests that the effort exerted when undertaking the exit VO_2_max was similar between groups.

The time saving benefits of high intensity activity have received much emphasis. The time saved by those adhering to the AIT exercise prescription was however modest, when compared to the duration of the exercise sessions for WALK. This was in part because the recovery phase of the interval training (3 minutes) was quite long. It may be possible to shorten the rest interval without any loss in fitness [30]. Another feature of the overall study design, incorporated to reflect best practice, was a warm up and cool down period totaling 15 minutes per exercise session. The optimal prescription for warm up and cool down is unknown [31]. Future studies may therefore be able to shorten both the recovery phase in AIT and MVIT exercise prescriptions and also the warm up and cool down phases undertaken by all participants, thereby undertaking shorter duration exercise sessions. This could hopefully be achieved without an increase in injury or similar harms.

As discussed above, extending observations from the controlled environment of the exercise laboratory to a health outcomes effectiveness study in a real world community setting, inevitably increases study complexity [32]. One aspect of this increased study complexity relates to non-adherence, which in this study was due to a mix of illness and injury, as well as behaviorally related reasons for non-adherence (see Figure 1). How best to analyze a study when some participants will inevitably be unable or unwilling to adhere to the exercise prescription or complete the exercise program? The usual approach to the problems of non- adherence and missing outcome data is to undertake an ITT analysis and this has traditionally been seen as the most conservative of approaches. This view has however been challenged and an alternative approach that includes information on adherence and efficacy has been advocated [33]. Details of both ITT and PP analyses, as well as details on study adherence, are therefore included in the current paper.

### Features that may limit the generalizability of this study

The screen failure rate was high but was similar to that of other exercise studies using community recruitment [34],[35]. The screening process did not however impede recruitment of a high proportion of participants with chronic health problems, as judged by the number of participants with a positive response to the PAR-Q (physical activity readiness questionnaire) and high percentage use of long term medications. Participants were asked not to change their diet during the study, to better study the effect of a single type of intervention, however a dual strategy of combined exercise and dietary interventions are of greater health benefit than either intervention alone and as such form the basis for the majority of evidence based healthy lifestyle recommendations. Finally, although the higher intensity activity groups in the current study reported a trend to increased injury compared to walking, the adverse outcomes in this study may in part reflect site specific features such as the quality of both the training surface and also artificial illumination.

### Additional limitations of this study

Undertaking parallel contemporaneous interventions in a group setting resulted in multiple participants exiting the study at the same time, which in turn produced logistic challenges for both the participants and the assessors in relation to obtaining exit assessments, immediately after the last exercise session. Results from the current study are therefore likely to reflect chronic rather than acute metabolic changes that might occur following the introduction of exercise.

In summary, this feasibility study's small change in VO_2_max contrasts to that seen in previous HIIT studies and highlights aspects of study methodology that, whilst they have been shown to work well in laboratory based efficacy studies, appear to work less well in a real world environment. Consideration should therefore be given to modifying these aspects of study design in future studies. As an example, use of submaximal VO_2_max assessment, rather than the VO_2_max assessment as used in the current study, might allow participants with minor illness or injury that precludes exercising to maximal intensity, to complete an exit assessment. Some participants in the current study experienced minor exercise related injuries (see Table S1) and were therefore asked to reduce exercise intensity for a short time, thus their adherence to the exercise intervention was reduced. Minor injuries may have been minimised by the use of a different exercise venue, a more gradual increase in training intensity and also a greater variation in activities. In laboratory based efficacy studies, behavioural support is usually minimal [32]. Behavioural support in the current study was modest when taking into account participants' history of inactivity. The challenge for future effectiveness studies aimed at determining how HIIT is best used in real world settings therefore relates not just to the details of the exercise prescription, but to the retention of important real world elements of study design such as low cost and recruitment of at risk participants, whilst at the same time aiming for a high rate of adherence to the intervention.

### Conclusions

This community based feasibility study extends the findings of earlier studies by showing that it is possible when undertaking high intensity interval training in a real world setting, to improve cardiorespiratory fitness in overweight, inactive participants and at the same time reduce the duration of the exercise sessions, compared to walking. The overall change in VO_2_max was however less than the changes previously observed in more structured research settings. In the real world setting of the current study, non-adherence to the exercise program is likely to be the main reason for the small observed changes in VO_2_max.

## Supporting Information

Checklist S1 CONSORT checklist.
(DOC)

Protocol S1 Trial Protocol.
(DOC)

Table S1Number (description) of participants experiencing an adverse event by exercise group allocation. ^a^Event of sufficient severity that participant exited study early. ^b^Event of sufficient severity that participant did not undertake exit 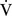
O_2max_.(DOCX)

Text S1 Ancillary methodological details.
(DOCX)
