# Correction: High Intensity Interval Training in a Real World Setting: A Randomized Controlled Feasibility Study in Overweight Inactive Adults, Measuring Change in Maximal Oxygen Uptake

**DOI:** 10.1371/journal.pone.0092651

**Published:** 2014-03-18

**Authors:** The PLOS ONE Staff

The main study outcome “VO_2_max” appears with incorrect formatting in these three instances: The first sentence of the Results portion of the Abstract, the second sentence of the second paragraph of the Results section, and the Table S1 description in the Supporting Information section.

## References

[1] Lunt H, Draper N, Marshall HC, Logan FJ, Hamlin MJ, et al (2014) High Intensity Interval Training in a Real World Setting: A Randomized Controlled Feasibility Study in Overweight Inactive Adults, Measuring Change in Maximal Oxygen Uptake. PLoS ONE 9(1): e83256 doi:10.1371/journal.pone.0083256 24454698 10.1371/journal.pone.0083256 PMC3890270

