# Supplementary material for: High Intensity Interval Training in a Real World Setting: A Randomized Controlled Feasibility Study in Overweight Inactive Adults, Measuring Change in Maximal Oxygen Uptake
Source: PLoS One. 2014 Jan 13;9(1):e83256. doi: 10.1371/journal.pone.0083256 (PMC3890270; doi:10.1371/journal.pone.0083256)
Supplement: Protocol S1 — Trial Protocol. (DOC) [file pone.0083256.s002.doc]

# NATIONAL APPLICATION FORM FOR ETHICAL APPROVAL OF A RESEARCH PROJECT

NAF-2009-v1

The application guidelines (NAFG-2009-v1) are to be read before completing this form to ensure that the questions are answered appropriately.

The electronic version of this form is formatted the same way as the paper version so that, for example, where an answer needs six lines, six lines are formatted, but where an answer only needs one line, one line is formatted. Please note the number of lines allowed for a question before answering it and make sure that you do not use extra lines.

You may find it helpful to print out the application form before completing it to help you to keep to the page limits allowed. **No extra pages should be added,** except where specified, as appendices.

The relevant paragraphs of the Operational Standard for Ethics Committees (Ministry of Health document) have been included in subject headings for reference.

The page breaks are not to be deleted as this will affect the formatting of the form.

When collating your application, please ensure that the information sheet, consent form and any attachments are placed behind the application form before copying. Applications not correctly collated, ie not in complete sets ready to be sent to committee members will be returned.

**Do not include this page with your application.**

# Checklist for Applicants – attach to front of application

**Before sending your application form, please check to make sure that all relevant information has been attached. If not applicable to the application write N/A. Protocols, information sheets, consent forms, questionnaires, advertisements, letters of invitation, data collection or other study forms must have a version number and date (marked *).**

**Please note: Incomplete applications will not be considered. Pending is an option only for written confirmation of Maori consultation, SCOTT approval, and Locality assessment by organisation. For multi-region studies, the documentation for one site must be complete.**

| **Reference** | **Item** | **Yes, pending or N/A** |
| --- | --- | --- |
| Observational Studies Guidelines 5.11 | * Study protocol – must be supplied with all applications | Yes |
| Page 21 of NAFG, QE on NAF | * Consent form | Yes |
| Page 23 of NAFG, QE on NAF | *Information sheet | Yes |
| QA5.4 on NAF | * Questionnaire/interview guidelines | Yes |
| Page 8 of NAFG, QA2 of NAF | Scientific assessment | Yes |
| QA5.3 of NAF | Statistical report | Yes |
| QD2, NAFG page 8 Q A4 | * Advertisement, letter of invitation | Yes |
| Section F of NAFG, Page 15 | Evidence of Māori consultation | Pending |
| Part 4 of NAF | Declaration signed by principal investigator, Head of Department or Dean (for each site) | Yes |
| Part 4, Form A or B of NAF | Accident compensation declaration correctly witnessed | N/A |
| NAFG page 20, NAF page 28 | Form/s for registered and unregistered medicines | N/A |
| Pages 11 and 35 (Appendix 1) of NAFG, QB17 on NAF | Standing Committee on Therapeutic Trials (SCOTT) approval attached if drug is unregistered in New Zealand | N/A |
| Locality assessments form(s) NAFG pages 18-20 | Completed by ethics committee if required, or completed by locality organisation(s) if received at time of submission | Pending |
| NAFG pages 30-32 | Part 5: If there any use of tissue (includes blood, saliva, skin) | Yes |
| Appendix 2 of NAFG | Part 6: If the research involves any gene or genetic studies | Yes |
| Appendix 2 of NAFG | Part 7 if the study involves xenotransplantation | N/A |
| NAFG pages 33-34 | Part 8 if any participants are unable to consent themselves including children | N/A |
| Parts 6 and 7, Appendix 2 of NAFG | GTAC approval if required | N/A |
| QB17 of NAF, Appendix 4 of NAFG | National Radiation Laboratory risk assessment if required | N/A |
| **Company sponsored studies** | Investigator brochure (if product is unregistered in New Zealand) | N/A |
|  | Signed indemnity agreement (sponsor/institution/investigator) |  |
|  | Current company insurance certificate |  |
| If yes to C.6 | Evidence of sponsor indemnity insurance to cover C.6 |  |
| If yes to C.6 | Evidence of hospital/institution indemnity insurance to cover C.6 |  |
| If yes to C.6 | Evidence of Investigator indemnity insurance to cover C.6 |  |

# NATIONAL APPLICATION FORM FOR ETHICAL APPROVAL OF A RESEARCH PROJECT

| Ethics reference number and date received (for office use only) |
| --- |

# Part 1: Basic Information

| 1. Full project title (include protocol number if applicable) | | | | | | | | | | | | |  |
| --- | --- | --- | --- | --- | --- | --- | --- | --- | --- | --- | --- | --- | --- |
|  | Health Benefits of high intensity exercise for populations at risk of diabetes and cardiovascular disease | | | | | | | | | | | | |
|  | | | | | | | | | | | | |  |
| 2. Short project title (lay title) | | | | | | | | | | | | |  |
|  | Health Benefits of High Intensity Exercise | | | | | | | | | | | | |
|  | | | | | | | | | | | | |  |
| 3. Principal investigator’s name and position | | | | | | | | | | | | |  |
|  | Dr Helen Lunt, Physician, CDHB | | | | | | | | | | | | |
|  | | | | | | | | | | | | |  |
| 4. Contact address of principal investigator | | | | | | | | | | | | |  |
|  | Christchurch Diabetes Centre  550 Hagley Avenue  Christchurch, 8011 | | | | Work phone no. | | xxxxxxx | | | | | | |
|  |  |  |  |  | Emergency no.* | | xxxxxxx | | | | | | |
|  |  |  |  |  | Fax | | 03 364 0171 | | | | | | |
|  |  |  |  |  | Email | | helen.lunt@cdhb.govt.nz | | | | | | |
|  | | | | | | | | | | | | |  |
| 5. Principal investigator’s qualifications and experience in the past five years (relevant to proposed research) | | | | | | | | | | | | |  |
|  | MB ChB, FRACP, DM  Physician; Christchurch Diabetes Centre, 1993 to present.  Clinical Senior Lecturer; Christchurch School of Medicine, 1993 to present.  Investigator and co-investigator to numerous successfully completed diabetes-related studies including multinational clinical trials. | | | | | | | | | | | | |
|  | | | | | | | | | | | | |  |
| 6. Co-investigator’s name(s), qualifications and position(s) and,. if more than one locality; principal investigator at **each** locality | | | | | | | | | | | | |  |
|  | A | Dr. Nick Draper, PhD, Senior Lecturer, University of Canterbury (Principle Investigator at Uof C locality). | | | | | | | | | | | |
|  | B | Dr. Helen Marshall, (PhD), Research Assistant, University of Canterbury. | | | | | | | | | | | |
|  | C | Florence Logan, (Research Nurse), Christchurch Diabetes Centre. | | | | | | | | | | | |
|  | D | Mary Martin-Smith (Public Health Nurse), Canterbury District Health Board. | | | | | | | | | | | |
|  | E | Associate Professor Mike Hamlin, (PhD), Lincoln University | | | | | | | | | | | |
|  | F | Dr. Jerry Shearman, (PhD), CPIT | | | | | | | | | | | |
|  | G | Jane Hardcastle, (Research Nurse), CPIT | | | | | | | | | | | |
| |  |  | | --- | --- | | | | | | | | | | | | | |  |
| 7.1 Address of A above | | | | | | | | | | | | |  |
|  | School of Sciences and Physical Education  University of Canterbury  Private Bag 4800  Christchurch, 8140 | | | | Work phone no. | | xxxxxxx | | | | | | |
|  |  |  |  |  | Emergency no.* | |  | | | | | | |
|  |  |  |  |  | Fax | |  | | | | | | |
|  |  |  |  |  | Email | | nick.draper@canterbury.ac.nz | | | | | | |
|  | | | | | | | | | | | | |  |
| 7.2 Address of B above | | | | | | | | | | | | |  |
|  | School of Sciences and Physical Education  University of Canterbury  Private Bag 4800  Christchurch, 8140 | | | | Work phone no. | | xxxxxxx | | | | | | |
|  |  |  |  |  | Emergency no.* | |  | | | | | | |
|  |  |  |  |  | Fax | |  | | | | | | |
|  |  |  |  |  | Email | | Helen.marshall@canterbury.ac.nz | | | | | | |
|  | | | | | | | | | | | | |  |
| 7.3 Address of C above | | | | | | | | | | | | |  |
|  | Christchurch Diabetes Centre  550 Hagley Ave  Christchurch | | | | Work phone no. | | xxxxxxx | | | | | | |
|  |  |  |  |  | Emergency no.* | | xxxxxxx | | | | | | |
|  |  |  |  |  | Fax | | 03 3640171 | | | | | | |
|  |  |  |  |  | Email | | Florence.logan@cdhb.govt.nz | | | | | | |
|  | | | | | | | | | | | | |  |
| 7.4 Address of D above | | | | | | | | | | | | |  |
|  | Community and Public Health  Canterbury District Health Board  P O Box 1475  Christchurch, 8140 | | | | Work phone no. | | xxxxxxx | | | | | | |
|  |  |  |  |  | Emergency no.* | |  | | | | | | |
|  |  |  |  |  | Fax | |  | | | | | | |
|  |  |  |  |  | Email | | mary.martin-smith@cdhb.govt.nz | | | | | | |
|  | | | | | | | | | | | | |  |
| 7.5 Address of E above | | | | | | | | | | | | |  |
|  | Department of Social Science, Parks, Recreation, Tourism and Sport  P O Box 84, Lincoln University  Lincoln, 7647 | | | | Work phone no. | | xxxxxxx | | | | | | |
|  |  |  |  |  | Emergency no.* | |  | | | | | | |
|  |  |  |  |  | Fax | | 03 325 3857 | | | | | | |
|  |  |  |  |  | Email | | mike.hamlin@lincoln.ac.nz | | | | | | |
|  | | | | | | | | | | | | |  |
| 7.6 Address of F above | | | | | | | | | | | | |  |
|  | School of Applied Sciences and Allied Health  CPIT  P O Box 540  Christchurch, 8015 | | | | Work phone no. | | xxxxxxx | | | | | | |
|  |  |  |  |  | Emergency no.* | |  | | | | | | |
|  |  |  |  |  | Fax | |  | | | | | | |
|  |  |  |  |  | Email | | shearmanj@cpit.ac.nz | | | | | | |
|  | | | | | | | | | | | | |  |
| 7.7 Address of G above | | | | | | | | | | | | |  |
|  | School of Applied Sciences and Allied Health  CPIT  P O Box 540  Christchurch, 8015 | | | | Work phone no. | | xxxxxxx | | | | | | |
|  |  |  |  |  | Emergency no.* | |  | | | | | | |
|  |  |  |  |  | Fax | | xxxxxxx | | | | | | |
|  |  |  |  |  | Email | | HardcastleJ@cpit.ac.nz | | | | | | |
|  |  | | | |  | | | | | | | | |
|  | | | | | | | | | | | | |  |
|  | | |  | | |  | | | | |  | | |
|  |  |  |  |  |  |  |  |  |  |  |  |  |  |
|  |  |  |  |  |  |  |  |  |  |  |  |  |  |
|  |  |  |  |  |  |  |  |  |  |  |  |  |  |
|  | | | | | | | | | | | | |  |
|  | | | | | | | | | | | | |  |
| 8. Where this is supervised work | | | | | | | | | | | | |  |
| 8.1 Supervisor’s name | | | | Not applicable | | | | | | | | |  |
| Position | | | |  | | | | | | | | |  |
| Daytime phone number | | | |  | | | | | | | | |  |
|  | | | | | | | | | | | | |  |
| 8.2 Signature of supervisor (where relevant)  Declaration: I take responsibility for all ethical aspects of the project | | | |  | | | | | | | | |  |
|  | | | | | | | | | | | | |  |
| 9. List locality organisation/s involved, including contact address, and complete the locality assessment in Part 4: Declarations (refer to the Guidelines (NAFG-2009-v1)) | | | | Christchurch Diabetes Centre, 550 Hagley Ave, Christchurch.  School of Sciences and Physical Education, University of Canterbury, Private Bag 4800, Christchurch. | | | | | | | | |  |
|  | | | | | | | | | | | | |  |
| 10. I wish the protocol to be heard in a closed meeting. | | | | | | | |  | Yes | X | | No |  |
|  | | | | | | | | | | | | |  |
| If the answer is yes, please provide a reason why you wish the protocol to be heard in a closed meeting in accordance with the Official Information Act 1982. | | | |  | | | | | | | | |  |
|  | | | | | | | | | | | | |  |
| 11. If the study is based, in part or in full, overseas, which countries are involved? | | | | Not applicable | | | | | | | | |  |
|  | | | | | | | | | | | | |  |
| 12. Has this application been reviewed by another ethics committee in New Zealand | | | | | | | |  | Yes | X | | No |  |
| or overseas? | | | | | | | | | | | | |  |
| (If yes, advise which country, the name of the committee/s and the decision/s of the committee/s)  Please note a copy of the report/s may be requested. | | | |  | | | | | | | | |  |
|  | | | | | | | | | | | | |  |
| 13. **Human tissue** – Does the project involve collection or use of human | | | | | | | | X | Yes |  | | No |  |
| tissue? If **yes**, complete Part 5. | | | | | | | | | | | | |  |
|  | | | | | | | | | | | | |  |
| 14. **Gene studies** – Does this research involve any gene or genetic studies? | | | | | | | |  | Yes | X | | No |  |
| If **yes**, complete Part 6. | | | | | | | | | | | | |  |
|  | | | | | | | | | | | | |  |
| 15. **Xenotransplantation** – Does this research involve the transplantation of | | | | | | | |  | Yes | X | | No |  |
| living biological material from one species to another? | | | | | | | | | | | | |  |
| If **yes**, complete Part 7. | | | | | | | | | | | | |  |
|  | | | | | | | | | | | | |  |
| 16. **Consent** – Are all participants able to provide consent for themselves? | | | | | | | | X | Yes |  | | No |  |
| If **no**, complete Part 8. | | | | | | | | | | | | |  |
|  | | | | | | | | | | | | |  |
| 17. **Lay summary** – give a brief **lay (non-technical)** summary of the study (not more than 200 words) such as you would give as an explanation to participants. | | | | | | | | | | | | |  |
|  | Traditionally low intensity “fat burning” endurance training (LIET), for example steady walking or jogging, has been prescribed for those at-risk of developing type 2 diabetes and cardiovascular disease (CVD). This type of exercise has resulted in some reduction of risk factors but limited exercise adherence success. Recent research suggests high intensity exercise (HIIT) or maximal volitional intensive training (MVIT), which is a form of interval training, may provide a time efficient method for improving risk factor reduction. This reduction in time input may help with exercise adherence. Much of the HIIT research has been based in the exercise laboratory. The study objective is to examine whether these findings can be translated to real world settings and populations at-risk of diabetes and cardiovascular disease, in a New Zealand context. In the first 12 week phase of the research translation, standard low intensity activity (LIET) will be compared to two high intensity programmes (HIIT and MVIT), in at-risk populations. In the second 6 month phase the high intensity exercise interventions will be adapted to match those readily available to participants in their local area. Changes in fitness and metabolic risk factors will be assessed along with exercise adherence. | | | | | | | | | | | |  |
|  | | | | | | | | | | | | |  |
| 18. Proposed starting date (dd/mm/yy) | | | | 01/02/2010 | | | | | | | | |  |
|  | | | | | | | | | | | | |  |
| 19. Proposed finishing date (dd/mm/yy) | | | | 30/07/2011 | | | | | | | | |  |
|  | | | | | | | | | | | | |  |
| 20. Duration of project in New Zealand (mm/yy) | | | | 18 months | | | | | | | | |  |
|  | | | | | | | | | | | | |  |
| 21. Proposed final report date (mm/yy) | | | | 01/12 | | | | | | | | |  |
|  | | | | | | | | | | | | |  |
| 22. Has the clinical trial been registered? | | | | | | | |  | Yes | X | | No |  |
|  | | | | | | | | | | | | |  |
| If **yes**, name the register. | | | |  | | | | | | | | |  |
|  | | | | | | | | | | | | |  |
| If **no**, has registration been applied for? | | | | | | | |  | Yes | X | | No |  |
|  | | | | | | | | | | | | |  |
|  | **Comment**: The investigators plan to register the study with the Australian New Zealand Clinical Trials registry, once Ethical approval has been obtained (ie following any suggested amendments from the Ethics Committee) | | | | | | | | | | | | |
|  | | | | | | | | | | | | |  |

# Part 2: Ethical Principles

## A. Validity of research *(Operational standard paragraphs 53–59)*

### Scientific basis

#### A1. Aims of the project

| A1.1 What is the hypothesis/research question(s) and/or the specific aims of the project? (State briefly.) | |
| --- | --- |
|  | **Comment**: This translational research project aims to assess whether the beneficial effects of high intensity exercise programmes seen in controlled settings e.g. the exercise laboratory, can be reproduced in a ‘real world’ New Zealand setting, in subjects at risk of diabetes and CV disease. VO2max is a positive predictor of beneficial health outcomes,^1^ and change in VO2max after exercise will be used as the primary endpoint.  The study will be undertaken in two phases. Both phases will follow standard RCT design. Phase 1 is predominantly an efficacy study, aimed to test the hypothesis that high intensity activity produces results that are superior to that of a standard walking programme. Subjects at risk of diabetes and cardiovascular disease will be recruited from the community, by advertisement. They will be randomised to one of the following: control intervention (LIIT) ie the usual exercise ‘prescription’ and two comparator exercise interventions, high (HIIT) intensity activity or maximal volitional intensity training (MVIT) ie interval training. Phase 2 is predominantly an effectiveness study. The most effective high intensity exercise prescription from Phase 1 will be compared with LIIT. Once again, the programme will be offered to subjects recruited from the community, by advertisement and also by word of mouth. Recruitment for Phase 2 will be weighted towards Maori participation. The primary outcome for both phases of the study is change in subjects’ fitness, as assessed by changes in VO2max. Changes in metabolic risk factors will be used as secondary outcomes.  *1. Kodama et al, Cardiorespiratory fitness as a quantitative predictor of all-cause mortality and cardiovascular events in healthy men and women: A meta-analysis. JAMA 2009: 301: 2024-2035.* |

#### A2. Scientific background of the research

| A2.1 Has this project been scientifically assessed by independent review? | | | X | Yes |  | No |
| --- | --- | --- | --- | --- | --- | --- |
|  | | | | | | |
| If **yes**, describe the process, for example, HRC funding assessment process. *A copy of the report should also be attached. The researcher’s response may also be included.* | | This project has been through the DHB/HRC scientific assessment process, as part of the DHB research fund: Translational research in CV diabetes, diabetes and obesity. See the following website for details, including funding details:  http://dhbrf.hrc.govt.nz/index.php/current_projects | | | | |
|  | | | | | | |
| If **no**, do you intend to have the project scientifically assessed and by whom? | |  | | | | |
|  | | | | | | |
| A2.2 Describe the scientific basis of the project **(300 words maximum**). Where this space is inadequate, continue on a separate sheet of paper. *Do not* delete page breaks or renumber pages. | | | | | | |
|  | Physical inactivity is endemic in developed societies and ‘lack of time’ is cited as a major contributor to inactivity (1, 2). The usual exercise prescription given for those with, or who are at-risk of, type 2 diabetes and cardiovascular disease is high volume low intensity endurance training (LIET). Recently low volume, high intensity interval training (HIIT) has been proposed as a more time efficient strategy for improving oxidative capacity and inducing metabolic adaptations equal to LIET. This approach has successfully been used with obese ‘middle aged’ individuals (3, 4). We will examine the health and exercise adherence benefits of HIIT or MVIT in comparison with LIET, in patients who are diagnosed with risk factors associated with the development of type 2 diabetes and CVD.  1. Blair SN. Physical inactivity: the biggest public health problem of the 21st century. BMJ. 2009;43(1):1-2.  *2. Godin GR, Desharnais P, Valois P, Lepage P, Jobin J, Bradet R. Differences in perceived barriers to exercise between high and low intenders: Observations among different populations. Am J Health Promot. 1994;8:279-85.*  *3. Tjonna AE, Lee SJ, Rognmo O, et al. Aerobic interval training versus continuous moderate exercise as a treatment for the metabolic syndrome: a pilot study. Circulation. 2008;118(4):346-54.*  *4. Wisloff U, Stoylen A, Loennechen JP, et al. Superior cardiovascular effect of aerobic interval training versus moderate continuous training in heart failure patients: a randomized study. Circulation. 2007;115(24):3086-94.* | | | | | |

#### A3. Study design

| A3.1 Describe the study design. Where this space is inadequate, continue on a separate sheet of paper. *Do not* delete page breaks or renumber pages. | |
| --- | --- |
|  | The study follows a randomised controlled trial (RCT) design and is divided into two Phases. Phase 1 is primarily an efficacy study, conducted over 12 weeks. This study will examine three exercise prescriptions (LIIT, MVIT, HIIT). Phase 2 is an effectiveness study, will be weighted towards Maori participation and will be conducted over 6 months. The exercise prescriptions used in Phase 2 will be the most successful high prescription from Phase 1 (ie either MVIT or HIIT), compared to a standard low intensity walking programme (LIIT) .The exercise prescription for the LIIT, MVIT and HIIT will be isocaloric ie those taking part in the high intensity arm of the programme will spend less overall time undertaking their exercise programme. Participants will have an elevated BMI and will not be regular exercisers. Participants will be asked to follow an exercise prescription as part of the study but will not be asked to change their diet. Participants must be physically well enough to able to undertake walking as their main form of exercise. Each phase will have separate participants.  All three of the exercise interventions will have a walking based component. They will be conducted by exercise leaders, trained to undertake the relevant exercise programmes in a standardised manner. (These exercise leaders will be provided by CPIT and are likely to include applied exercise physiology students undertaken a supervised Practicum). The exact intensity of exercise will however be individualised ie based on the subject’s pre-study fitness levels and performance when undertaking maximal volitional exercise. Fitness will improve during the study, thus exercise load will increase as the study progresses. Some subjects in the high intensity arm may therefore be considered ‘fit’ enough to incorporate a light jogging component to their exercise plan.  Data collection for Phase 1 will be undertaken before and after the twelve week intervention period. The same data will be collected for Phase 2 but will be collected at three time points: week 0, week 13 and week 26. Data will include a standardised exercise/fitness assessment, measuring V02 max on a treadmill test, standard anthropomorphic measurements, including waist circumference and an impedance measurement of total body fat, fasting venous blood collection for measurement of insulin, glucose, lipids and HsCRP. Subjects will complete several validated questionnaires including a four day food diary (to assess the extent that change in diet might act as a confounder) and quality of life assessments. The exercise physiology researchers undertaking the primary outcome measurement (VO2 max), will be blinded to group allocation.  Trial conduct, including the reporting of subject enrolment and follow up, will be undertaken using the CONSORT (Consolidated Standards of Reporting Trials) framework.  In summary phase 1 will look to use the positive results found in laboratory based heavily structured and tightly monitored exercise interventions and transfer these positive results to more community-based, less structured but still tightly controlled exercise intervention. In Phase 2 of the study we want to use the results found in phase 1 and apply them to an even less-structured, less tightly controlled but more realistic and much less expensive community-based exercise intervention.  (see Appendix 1 on separate sheet for more detail). |

#### A4. Participants

| A4.1 How many participants do you intend to recruit? (Include details for each locality organisation.) | |
| --- | --- |
|  | Phase 1;- 60 participants, anticipating that 48 will complete the study (16 in each arm).  Phase 2;- This less structured community based phase of the study is likely to experience a higher ‘drop out’ rate. 60 participants will be recruited, anticipating that 44 participants will complete the study (22 in each arm). |
|  | |
| A4.2 Give a justification for the number of research participants proposed, giving the details of power calculations when appropriate. | |
|  | A sample size of 16 in each group for the first study, will enable effect sizes >1.0 to be detected as statistically significant (two tailed-alpha=0.05) with 80% power. Assuming an untrained VO2 max of 30 ± 4ml/kg/min, this equates to differences in changes in VO2 max over 12 weeks of approximately 4 ml/kg/min.  A sample size of 22 per group in the second study will enable effect sizes >0.9 to be detected as statistically significant (two tailed-alpha=0.05) with 80% power, this equates to differences in changes in VO2max over 26 weeks of approximately 3.5ml/kg/min.  (To place this in context, a sedentary individual typically has a VO2 max of 25-35ml/kg/min +/- SD of 4 and a trained adult has a VO2 max around 40-60 ml/kg/min). |
|  | |
| A4.3 If randomisation is used, explain how this will be done. | |
|  | After consenting and baseline measurements, subjects will be randomly allocated in a 1:1:1 ratio to the exercise interventions, under the supervision of the medical biostatistician (CF).For both studies it is important to achieve a balance in gender and this will be achieved by stratified randomisation. |
|  | |

#### A5. Statistical method

| A5.1 Is the method of analysis quantitative? | | | X | Yes |  | No |
| --- | --- | --- | --- | --- | --- | --- |
|  | | | | | | |
| Or qualitative? | | |  | Yes | X | No |
|  | | | | | | |
| If the method of analysis is **wholly qualitative**, go to question A5.4. If the method of analysis is **wholly or partly quantitative**, complete the following: | | | | | | |
|  | | | | | | |
| A5.2 Describe the statistical method that will be used to analyse the data. | | | | | | |
|  | Initial data analysis will be conducted by Dr Nick Draper and colleagues using the Statistical Package for the Social Sciences (SPSS) software programme. Final data analysis will be undertaken under the supervision of Associate Professor Chris Frampton, medical biostatistician. For both studies changes in continuous outcome measures (e.g. VO2max, BMI, etc) will be compared between groups using ANCOVA. These models will utilise the stratification variable, gender, as a factor and the baseline measure as a covariate. The appropriateness of these parametric analyses will be confirmed by visual inspection of the residual plots. If these do not confirm normality, data maybe transformed prior to analyses and if this does not remedy problems then non-parametric analyses will be undertaken. A two-tailed p value of <0.05 will be taken to indicate statistical significance.  The primary outcome measure is change in VO2max.  The secondary outcome measures are changes in: BMI, waist circumference, blood pressure, insulin sensitivity (HOMA) and lipids.  Tertiary outcome measures are changes in: % body fat as measured by bioimpedance, Quality of Life, hs CRP.  Samples will also be collected for an exploratory study measuring changes in a novel marker of muscular ‘fitness’, namely monocyte mitochondrial load. | | | | | |
|  | | | | | | |
| A5.3 Has specialist statistical advice been obtained about this study? | | | X | Yes |  | No |
|  | | | | | | |
| If **yes**, from whom? (A brief statistical report should be included if appropriate.) | | Associate Prof Chris Frampton, University of Otago. | | | | |
|  | | | | | | |
| A5.4 If the method of analysis is **wholly or partly qualitative**, specify the method. Why is this method appropriate? If interviews are to be used, include the general areas around which they will be based and a copy of the interview guide, if one is to be used. Copies of any questionnaires that will be used must be included. | | | | | | |
|  | Not applicable | | | | | |

#### A6. Expected outcomes or impacts of research

| A6.1 What is the potential significance of this project for improved health outcomes? | |
| --- | --- |
|  | Evidence exists showing that high-volume LIET benefits fitness, health, and exercise adherence but this form of traditional training is usually undertaken by a narrow band of healthy participants of average or above average fitness. There is a paucity of evidence available to demonstrate whether traditional programs are appropriate for the wider community. Therefore, there is a clear need to examine outcomes and adherence to alternative exercise programmes such as a high intensity (low volume) exercise prescription. The potential benefits include decreased risk of developing cardiovascular disease and diabetes in at risk subjects, decreased risk of developing complications in those with pre-existing disease and improved quality of life. |
|  | |
| A6.2 What is the potential significance of this project for the advancement of knowledge? | |
|  | Peak aerobic fitness has been shown to be the strongest independent predictor of mortality compared with other established risk factors in healthy and unhealthy subjects. Previous research has found that HIIT improves aerobic fitness more than LIET. Moreover, interval training results in superior improvements in insulin signalling (in fat and muscle) and skeletal muscle biogenesis as well as reducing blood glucose levels and lipogenesis in adipocytes. It has also been found that HIIT can also benefit remodelling of heart muscle. However, prominent researchers examining this new exercise paradigm have stressed the need for further research with a wider range of populations. In addition, this knowledge needs to be applied to community-based interventions. Only through such research will it be possible to develop evidence-based alternatives to traditional time intensive high-volume exercise prescriptions for non-athletic populations. |
|  | |
| A6.3 What steps will be taken to disseminate the research results? | |
|  | A report will be prepared for the sponsors (DHB translational research fund, HRC). In addition, results of the project will be presented at various forums including national and international conferences and local seminars. Finally a number of peer-reviewed research articles will be prepared from the results of this project.  Study results will be fed back to local participants in a group setting, under the guidance of local Maori leaders. |

#### A7. Publication of results

| Will any restriction be placed on publication of results? | |  | Yes | X | No |
| --- | --- | --- | --- | --- | --- |
|  | | | | | |
| If **yes**, please supply details. |  | | | | |

#### A8. Funding

| A8.1 How will the project be funded? | | | | | |
| --- | --- | --- | --- | --- | --- |
|  | Funding for the main project has been granted through the 2009 Translational Research funding round, Health Research Council of New Zealand’s District Health Board Research Fund.  Separate funding will be sought for consumables for the mitochondrial depletion sub study (estimated cost $20,000). | | | | |
|  | | | | | |
| A8.2 Does the researcher, the host department, the host institution or the | |  | Yes | X | No |
| locality organisation have any conflict of interest, eg, financial interest, in the outcome of this research? If **yes**, please give details. | | | | | |
|  |  | | | | |

#### A9. Incentive payments

| A9.1 Have you read and understood the description of incentive payments in the | | X | Yes |  | No |
| --- | --- | --- | --- | --- | --- |
| Guidelines? | | | | | |
| **Note: Details about any payment (in money or kind) or reward made to participants recruited into the project are to be provided in question E10.** | | | | | |
|  | | | | | |
| A9.2 Does the funding available to the project depend upon the number of participants | |  | Yes | X | No |
| recruited, eg, is the funding on a per participant basis?. If **yes**, give details of the amount per participant. Where there is a significant difference between these, this incentive to recruit should be declared in the information sheet. | | | | | |
|  |  | | | | |
|  | | | | | |
| A9.3 Does the funding available to the project include any form of incentive (in money | |  | Yes | X | No |
| or kind) for the early or complete recruitment of a specified number of participants, eg, bonus payments to the researcher, host department or host institution? If **yes**, give details. | | | | | |
|  |  | | | | |
|  | | | | | |
| A9.4 Will **all** funding available to the project be passed through an audited research | | X | Yes |  | No |
| account or cost centre? If **yes**, give details. If **no**, specify why not. | | | | | |
|  | The project is registered with the Research Office, Christchurch Hospital. They will advise about the setting up of a Cost Centre, to ensure that funds are disbursed through an appropriate mechanism that will stand up to audit. | | | | |

## *B. Minimisation of harm (Operational standard p*aragraphs 60–68)

| B1. How many visits/admissions of participants will this study involve? Clarify what is in addition to standard treatment. Give also an estimate of total time involved for participants. | | | | | | | | | | | | |
| --- | --- | --- | --- | --- | --- | --- | --- | --- | --- | --- | --- | --- |
|  | **Phase 1:** In addition to the prescribed exercise sessions three times a week for <50 minutes, over 12 weeks (ie the intervention), participants will be required to attend one visit for consent, a fasting visit before and after completion of the study for fasting venesection and a non fasting visit to the exercise laboratory, before and after the completion of the study, for fitness testing and anthropomorphic measurements. Each of these 5 additional visits will take less than one hour.  **Phase 2:** The exercise treatment (ie intervention) for this phase is one supervised exercise training session and two unsupervised training sessions per week of up to 50 minutes duration per session, over a period of six months. After the consenting visit, subjects will attend for fasting venesection at weeks 0, 13 and 26 and attend the exercise laboratory for fitness testing and anthropomorphic measurements on a separate day at weeks 0, 13 and 26. Each of these 7 additional visits will take less than one hour. | | | | | | | | | | | |
|  | | | | | | | | | | | | |
| B2. Who will carry out the research procedures? | | | | | | | | | | | | |
|  | Venesection will be undertaken by trained researchers, including research nurses. Collection of anthropometric measurements will be undertaken by all researchers, under the supervision of Dr Jerry Shearman, who is Level II accredited with the **International Society for the Advancement of Kinanthropometry (ISAK).**  Clinical assessments will be undertaken by the research nurses, under the supervision of Dr Helen Lunt and also directly by Dr Lunt. Dr Lunt will be responsible for screening potential subjects’ medical fitness to participate in the project, in conjunction with a report from the subject’s General Practitioner, as required.  Exercise testing (ie measurement of V02max) will be undertaken by trained exercise physiology researchers – Dr. Nick Draper, Dr. Helen Marshall, Dr. Jerry Shearman, Dr. Mike Hamlin. The exercise interventions will be supervised either directly by these exercise physiologists, or indirectly through supervision of exercise physiology students, who will undertake the assessment. | | | | | | | | | | | |
|  | | | | | | | | | | | | |
| B3. What other research studies is the lead investigator currently involved with? | | | | | | | | | | | | |
|  | Dr Helen Lunt will have completed data collection for a three year HRC funded dietary study, by December 2009. She supervises sample collection on behalf of the Christchurch Diabetes Centre, for TrialNet, a long term multinational natural history study of antibody status of subjects at risk of type 1 diabetes. She is the Diabetes Centre site investigator for two international pharmaceutical studies of DPP IV inhibitors (an efficacy and a cardiovascular outcome study). | | | | | | | | | | | |
|  | | | | | | | | | | | | |
| B4. Where will the research procedures take place? | | | | | | | | | | | | |
|  | Fasting venous blood samples and questionnaires will collected at Christchurch Diabetes Centre.  Anthropometric and Exercise testing will take place at the Exercise Science Laboratory, University of Canterbury (Dovedale Ave Campus).  Exercise training will take place in a local park (e.g. Hagley Park) | | | | | | | | | | | |
|  | | | | | | | | | | | | |
| B5. How do the research procedures differ from standard treatment procedures? | | | | | | | | | | | | |
|  | Overweight subjects at risk of developing diabetes and cardiovascular disease do not usually have ‘free’ access to an exercise programme and fitness testing. The exercise programme and fitness testing will however follow a standardised protocol, which is similar to that undertaken by sports medicine physicians. Subjects will undergo venesection and complete questionnaires. | | | | | | | | | | | |
|  | | | | | | | | | | | | |
| B6. What are the benefits to research participants of taking part in the project? | | | | | | | | | | | | |
|  | Participants will benefit by learning about their own health and wellbeing. They are also likely to benefit by becoming healthier through a combination of physiological changes that occur following the introduction of regular exercise (e.g. weight loss, reduced blood pressure, improved lipid profile and lipid composition, increased insulin sensitivity). | | | | | | | | | | | |
|  | | | | | | | | | | | | |
| B7. Describe any methods for obtaining information. Attach questionnaires and interview guidelines. (If National Health Index (NHI) information is used, see the Guidelines (NAFG-2009-v1).) | | | | | | | | | | | | |
|  | Participants anthropometric and fitness data will be measured using standard tests and procedures. Information will also be gained via standard metabolic measurements (anthropomorphic measurements and blood tests). We will also gain information via the following validated, published questionnaires (see attachments).  Four questionnaires will be used. They are: 1. Four day prospectively collected food record, 2. Quality of Life questionnaire (SF36) 3. Fitness to exercise PAR-Q questionnaire and 4. readiness to change questionnaire. | | | | | | | | | | | |
|  | | | | | | | | | | | | |
| B8. Briefly describe the inclusion/exclusion criteria and include the relevant page number(s) of the protocol or investigator’s brochure. | | | | | | | | | | | | |
|  | **Inclusion Criteria:**  Aged 35-60 years inclusive  BMI ≥28 and ≤40  Sedentary (no regular physical activity in last 3 months).  **Exclusion Criteria:**  Underlying musculoskeletal conditions that preclude subjects being able to walk briskly for up to 50 minutes  Active cardiac conditions including angina  Intermittent claudication  Underlying respiratory conditions, including asthma requiring use of an inhaler within the previous one year  Any chronic condition which, in the opinion of the potential subject’s GP or principle investigator, would make it unsafe for the subject to participate in a high intensity exercise programme (ie brisk walking)  Myopathy, including subjects on statins with moderate to severe myopathic symptoms  Active psychiatric illness  Any other chronic condition that might require a change in medication during the study (for example subjects with diabetes on sulphonylureas and insulin, subjects requiring variable doses of oral corticosteroids)  Pregnant or planning to become pregnant during the duration of the study  History of malignancy (other than skin malignancy excluding melanoma) in the previous 5 years  Treated hypertension requiring more than one antihypertensive agent  Current or recent weight change (self reported) of >5kg in last 3 months  Other reasons why taking part would be practically difficult | | | | | | | | | | | |
|  | | | | | | | | | | | | |
| B9. What are the physical or psychological risks or side effects to participants or third parties? Describe what action will be taken to minimise any such risks or side effects. | | | | | | | | | | | | |
|  | **Physical risks to participants.** The PAR-Q questionnaire will be used to screen for higher risk participants with pre-existing minor health problems. Those with positive answers to health questions will be monitored more closely. As with any exercise programme, there is a risk of musculoskeletal injury but this will be minimised by subjects undertaking supervised warm-up exercises beforehand. Physical risks include unmasking of underlying asymptomatic cardiovascular problems during the fitness tests when participants will be asked to go up to their maximum exercise level. Physical risks will be minimised by strictly following exercise protocols. Trained individuals from either an exercise physiology or nursing/medical background with the relevant first aid qualifications will be available during the supervised exercise sessions. Appropriate emergency equipment and standardised emergency procedures are available at the exercise physiology laboratory.  **Psychological risks to participants.** There include anxiety prior to and during venesection and when completing fitness and anthropometric tests. Psychological risks associated with the fitness and anthropometric tests will be minimised by a variety of methods. These include undergoing fitness testing in secluded rooms away from other participants, receiving an explanation about the testing procedures and what is expected of the participants before starting the exercise testing. | | | | | | | | | | | |
|  | | | | | | | | | | | | |
| B10. What facilities/procedures and personnel are there for dealing with emergencies? | | | | | | | | | | | | |
|  | Venepucture will be undertaken by trained personnel ie research nurses and doctors, who are experienced in minimising and treating any adverse reaction associated with venepuncture, such as a vaso-vagal reaction (fainting). During exercise testing (to be undertaken at the University of Canterbury campus), the following staff/equipment will be present; a registered nurse, more than 3 people in the testing room at any time at least one of which will have current first aid certificate, defibrillator, direct line to campus Security, direct line to hospital. In addition, Security will be advised that the project is taking place in case of emergencies. | | | | | | | | | | | |
|  | | | | | | | | | | | | |
| B11. What arrangements will be made for monitoring and detecting adverse outcomes? | | | | | | | | | | | | |
|  | 1. **Exercise programme.** The direct supervisors of the exercise programme will ask participants to report minor (as well as major) physical symptoms, such as musculoskeletal pains. These adverse events will then report back to the research nurse and physician (Dr Helen Lunt). A course of action as a result of symptoms may be suggested e.g. a rest/recovery period away from exercise for minor musculoskeletal pains. Alternatively the participant may be advised to seek an opinion from their GP for more serious complains. 2. **Exercise testing.** Some subjects may suffer transient light headedness of breathlessness during maximal exercise testing. If this progresses, the exercise test will be terminated early. Participants will be observed for any adverse outcomes during the course of their exercise testing. After exercise testing participants will be monitored for at least 20 minutes. Exercise physiologists or their students will also be in regular contact with participants, as they will be supervising the regular exercise sessions. 3. **Venesection.** No major adverse events are anticipated but there may be minor bruising that will be minimised by applying pressure to the venepuncture site. Subjects with a history of vasovagal faints will be venesected lying down. | | | | | | | | | | | |
|  | | | | | | | | | | | | |
| B12. If the study is a clinical trial, are participants to be provided with a card | | | | | |  | Yes | | X | | No | |
| confirming their participation, medication and the contact phone number of the principal investigator? No, because they are not on any study medication | | | | | | | | | | | | |
|  | | | | | | | | | | | | |
| B12.1 Do you intend to inform the participant’s GP that their patient is a | | | | | | x | Yes | |  | | No | |
| participant in this study? (If yes, consent from the participant is required.) | | | | | | | | | | | | |
|  | | | | | | | | | | | | |
| B12.2 Do you intend to inform the GP of all clinically significant abnormal | | | | | | X | Yes | |  | | No | |
| results obtained during study conduct? | | | | | | | | | | | | |
|  | | | | | | | | | | | | |
| B13. Is the trial being reviewed by a data and safety monitoring board (DSMB)? | | | | | |  | Yes | | X | | No | |
|  | | | | | | | | | | | | |
| If **yes**, who is the funder of the DSMB? | | | |  | HRC |  | Sponsor | |  | | Other | |
|  | | | | | | | | | | | | |
| If **‘Other’**, please specify. | | Not applicable | | | | | | | | | | |
|  | | | | | | | | | | | | |
| B14. What are the criteria for terminating the study? | | | | | | | | | | | | |
|  | For individual participants, all adverse reactions will be reviewed by the research nurse and physician and advice may also be sought from the participant’s GP. Any of these health professionals may choose to terminate an individual’s involvement with the study, on medical grounds.  There are no-preset endpoints for terminating the study for protocol related reasons. | | | | | | | | | | | |
|  | | | | | | | | | | | | |
| B15. Will participants be exposed to any potential toxins, mutagens or | | | | | |  | Yes | | X | | No | |
| teratogens? | | | | | | | | | | | | |
| If **yes**, specify and outline the justification for their use. | |  | | | | | | | | | | |
|  | | | | | | | | | | | | |
| B16. Will any radiation or radioactive substances be used? | | | | | | |  | Yes | | X | | No |
| **Note: If any form of radiation is being used, please answer B16.1–B16.2. If no, go to question B17.** | | | | | | | | | | | | |
| B16.1 How many x-rays or other procedures are planned for the purposes of this study, ie, that are not part of standard treatment? | | |  | | | | | | | | | |
|  | | | | | | | | | | | | |
| B16.2 Under whose licence is the radiation being used? | | |  | | | | | | | | | |
|  | | | | | | | | | | | | |
| B16.3 Has the National Radiation Laboratory (NRL) risk assessment | | | | | | |  | Yes | |  | | No |
| Been completed? | | | | | | | | | | | | |
| If **yes**, please enclose a copy of the risk assessment and a contact name and phone number.  If **no**, please explain why not. | | |  | | | | | | | | | |
|  | | | | | | | | | | | | |
| B17. Will any medicines be administered for the purposes of this study? | | | | | | |  | Yes | | X | | No |
|  | | | | | | | | | | | | |
| B17.1 If **yes,** is Standing Committee on Therapeutic Trials (SCOTT) | | | | | | |  | Yes | |  | | No |
| approval required? | | | | | | | | | | | | |
| B17.2 Has SCOTT approval been given? (Please attach.) | | | | | | |  | Yes | |  | | No |
|  | | | | | | | | | | | | |
| B18. Does the study involve the use of health care resources? | | | | | | |  | Yes | | x | | No |
| If **yes**, please specify: | | | | | | | | | | | | |
|  | There is no direct use of health care resources. This study is sponsored by the DHB as part of a DHB/HRC translational funding grant. We will be utilising Diabetes Centre premises mainly outside clinic hours (early morning and weekends) and we will not be compromising clinical activities. CHL laboratory costs are fully funded as part of the study agreement. | | | | | | | | | | | |
|  | | | | | | | | | | | | |
| B19. What effect will this use of resources have on waiting list times for patients, that is, for diagnostic tests or for standard treatments? | | | | | | | | | | | | |
|  | Nil | | | | | | | | | | | |

## C. Compensation for harm suffered by participants *(Operational standard paragraphs 87–95)*

*(Refer also to Appendix 3 of the Guidelines (NAFG-2009-v1).)*

| C1. Will participants be treated by, or at the direction of, a registered health | | | | X | Yes |  | No |
| --- | --- | --- | --- | --- | --- | --- | --- |
| professional as part of the research? (Treatment includes screening, diagnosis, for definitions see the Guidelines (NAFG-2009-v1) pages 11-13.) | | | | | | | |
| If **no**, go to section D. If **yes**, please answer questions C2–C6.4. | | | | | | | |
|  | | | | | | | |
| C2. Is the research being carried out principally for the benefit of a | | | |  | Yes | X | No |
| manufacturer or distributor of the drug or item in respect of which the research is taking place? | | | | | | | |
| C2.1 If the answer to C2 is **yes**, please complete **Statutory Declaration Form B** and answer questions C3–C6.4. | | | | | | | |
| C2.2 If the answer to C2 is **no,** please complete **Statutory Declaration Form A** and go to section D. | | | | | | | |
|  | | | | | | | |
| Depending on all the circumstances, the minimum cover that is likely to be acceptable to the ethics committee is that provided under ACC. In any case, all exclusions to compensation must be clearly and explicitly set out in the participant information sheet, including those that may be described in C5. | | | | | | | |
| C3. Is the manufacturer/distributor’s agreement to provide compensation in | | | |  | Yes |  | No |
| accordance with the RMI attached? | | | | | | | |
|  | | | | | | | |
| C4. Has the manufacturer or distributor agreed to cover any injury/adverse | | | |  | Yes |  | No |
| consequence resulting from participation in this research? | | | | | | | |
| C4.1 If no, what qualifications have been specified for cover? | | | | | | | |
|  |  | | | | | | |
|  | | | | | | | |
| C4.2 **Limiting the type of compensation** | | | | | | | |
| C4.2.1 Has the manufacturer or distributor excluded any type of | | | |  | Yes |  | No |
| compensation, for example, pain and suffering, loss of earnings, loss of earning capacity, funeral costs, dependents’ allowances or any other financial loss or expenses? | | | | | | | |
| C4.2.2 If yes, please state what is excluded. (Include in the compensation statement on the information sheet) | | | | | | | |
|  | |  | | | | | |
|  | | | | | | | |
| C5. **Limiting liability – exclusion clauses** | | | | | | | |
| C5.1 Has the manufacturer or distributor limited or excluded liability if the | | | |  | Yes |  | No |
| injury is attributable to the negligence of someone other than the manufacturer or distributor (such as negligence by the investigator, research staff, the hospital or institution, or the participant)? | | | | | | | |
|  | | | | | | | |
| C5.2 Has the manufacturer or distributor limited or excluded liability if the | | | |  | Yes |  | No |
| if the injury resulted from a significant deviation from the study protocol by someone other than the manufacturer or distributor? | | | | | | | |
|  | | | | | | | |
| C5.3 Is evidence of the following indemnity insurance attached? | | | | | | | |
| Sponsor | | | |  | Yes |  | No |
|  | | | | | | | |
| If yes to either C5.1 or C5.2; | | | | | | | |
| Hospital/institution | | | |  | Yes |  | No |
|  | | | | | | | |
| Investigator | | | |  | Yes |  | No |
|  | | | | | | | |
| C5.4 Is company liability limited in any other way? | | | |  | Yes |  | No |
|  | | | | | | | |
| If **yes**, please specify. | | |  | | | | |

## *D. Privacy and confidentiality (Operational standard paragraphs 48–56*)

| D1. How will potential participants be identified? | Phase 1. Through advertisements in local newspapers, health magazines, radio and through local institutions e.g. CDHB and CPIT. Posters and fliers will be placed in relevant community venues, including GP surgeries.  Phase 2. As above, but the main initial recruitment will be through Whanau Tautoko Charitable Trust and their connections with local Maori Health Workers ie there will be some ‘word of mouth’ recruitment. |
| --- | --- |
|  | |
| D2. How will participants be recruited (for example, advertisements, notices)? | See answer to question D1. It is likely that Maori recruitment will be in part by word of mouth. |
|  | |
| D3. Where will potential participants be approached (for example, outpatient clinic)? If appropriate, describe by type (for example, students). | Participants will be asked to phone the research coordinator and the research coordinator will then approach the participant via phone, email or mail with further details of the study.  We will not directly recruit students or outpatients, as these subpopulations do not represent the ‘target’ population for the study. |
|  | |
| D4. Who will make the initial approach to potential participants? | The research study team will advertise the study (and in Phase 2 community based Maori health workers will facilitate the advertising by speaking directly to their clients). After initial contact to the research nurse by potential participants, the research nurse will then contact the potential participants with further details of the study. |
| NB: Do not include information on storage and use of tissue samples and related information in the following questions. That is covered separately under Part 5. | |
|  | |
| D5. How will data, including audio- and videotapes, be handled and stored to safeguard confidentiality (both during and after completion of the research project)? | Data will be collated by the principal investigators and sorted into appropriate areas of research. Information will be coded so that research data and participant identities are stored in separate places. For data analysis and reporting, participant identity will be strictly anonymised. All data (paper and e-data) will be held in medium to high security facilities. |
|  | |
| D6. What will be done with the raw data when the study is finished? | The raw data will be stored on an Excel spreadsheet on a secure network computer drive of the Canterbury District Health Board with a back-up on a similar drive at Canterbury University and on an appropriate storage medium safe-guarded by the Principal Investigator |
|  | |
| D7. How long will the data from the study be kept, and who will be responsible for their safe keeping? (Health information relating to an identifiable individual must be retained for at least 10 years, or in the case of a child, 10 years from the age of 16.) | The data will be stored indefinitely. The Principal Investigator will be responsible for the safe keeping of this information. |
|  | |
| D8. Name those who will have access to the raw data, participant information and/or clinical records during, or after, the study? | The Principal Investigator, and Dr. Nick Draper (Principal Investigator, University of Canterbury site) |
|  | |
| D9. Describe any arrangements to make results available to participants, including whether they will be offered their audio- or videotapes. | A summary of collated results will be offered to all participants |

## E. Informed consent *(Operational standard paragraphs 28–43)*

A participant’s informed consent should be obtained in writing, unless the procedures are not experimental and there are good reasons for not requiring written consent. If consent is not to be obtained in writing, the justification should be given and the circumstances under which consent is obtained should be recorded. Attach a copy of the information sheet and consent form provided to participants.

| E1. By whom, and how, will the project be explained to potential participants? | By the research nurses and researchers themselves (e.g. Dr. Helen Lunt, Dr. Nick Draper, Dr. Jerry Shearman, Dr. Helen Marshall, Dr. Mike Hamlin) | | | | |
| --- | --- | --- | --- | --- | --- |
|  | | | | | |
| E2. When and where will the explanation be given? | When participants are inquiring about the study after seeing the advertisement and before and during their consent visit. | | | | |
|  | | | | | |
| E3. Will a competent interpreter be available, if required? | |  | Yes | x | No |
|  | | | | | |
| If **no**, why not? | The supervised group exercise interventions will be undertaken by trained exercise physiology students, in English. To ensure that participants have a good understanding of exercise requirements, including safety aspects of training, all participants must therefore speak English. | | | | |
|  | | | | | |
| E4. How much time will be allowed for the potential participant to decide about taking part in the project? | Participants will be allowed as much time as they need to make a decision. | | | | |
|  | | | | | |
| E5. In what form (written, or oral) will consent be obtained? If oral consent only, state reasons. | Written. | | | | |
|  | | | | | |
| E6. If recordings are made, will participants be offered the opportunity to edit | |  | Yes |  | No |
| the transcripts of the recordings? Not applicable | | | | | |
|  | | | | | |
| E7. Will data or other information be stored for use in a different study for | |  | Yes | X | No |
| which ethics committee approval would be required? | | | | | |
| E7.1 If **yes**, please explain how. |  | | | | |
|  | | | | | |
| E8. Is there any special relationship between the participants and the researchers (for example, doctor/patient, student/teacher)? | No special relationships | | | | |
|  | | | | | |
| E9. Will there be any financial cost to the participant, for example, travel and parking costs? If so, will such cost be reimbursed? (Refer to the Guidelines (NAFG-2009-v1).) | Subjects will be given a petrol voucher to reimburse travel costs associated with the research visits (5 visits for Phase 1 and 7 visits for Phase 2), but not for the exercise training sessions | | | | |
|  | | | | | |
| E10. Will any payments be made to participants, or will they gain materially in | |  | Yes | X | No |
| other ways from participating in this project? | | | | | |
| E10.1 If **yes**, please supply details. |  | | | | |

## F. Cultural and social responsibility *(Operational standard paragraphs 73–82)*

Section F enshrines two fundamental principles. They are:

i. Culturally safe research practice: Research involving participants from specific ethnic or socially identified groups (even when small numbers from each group are involved) must involve those participant groups in the research process as full participants. Where a particular ethnic or socially identified group is the principal subject of the research, there must be engagement with appropriate parties, and this process must be outlined in the application.

ii. If the research is in an area of health inequalities, then the researcher must demonstrate how the research will contribute to achieving equity of outcomes for those population groups most in need within the public good health system.

| F1. Have you read the HRC booklet *Guidelines for Researchers on* *Health* | X | Yes |  | No |
| --- | --- | --- | --- | --- |
| *Research Involving Māori*? | | | | |

#### Relevance and responsiveness to Māori

| F2. **All health research conducted in Aotearoa New Zealand is of relevance to Māori**. How relevant is a decision to be made by Māori. The researcher must be able to articulate the context and the relevance of the proposed research to Māori and the possible consequences for Māori health outcomes, and generally, the greater the degree of relevance to Māori, the greater the expectation of participation of Māori and hence consultation expectations. | | | |
| --- | --- | --- | --- |
| F2.1 Given your approach to sampling, what are the anticipated numbers of Māori participants? | | | Phase 1 (efficacy study). This phase will be offered to the general population of Christchurch and will not specifically target Maori. Previous research would suggest that, using this general approach, Maori participation will be around 5% ie 3 of 60.  Phase 2 (effectiveness study). Sampling for this longer duration study will be focussed on and within the Maori community, in partnership with Maori Health workers. We are aiming for 50% of the participants being of Maori ethnicity (22 of 44 participants). |
|  | | | |
| F2.2 What is the incidence among Māori of the health issue/disability relevant to the study? | | | Modelling of incidence of type 2 diabetes would suggest that rates in Maori are around x3 those of the general population. Prevalence of one or more features of the metabolic syndrome is >80%, after age 40 years (*Simmons, Diabetes Care, 2004*). |
|  | | | |
| F3. Please explain how this research will contribute to improving Māori health outcomes and reducing health inequalities for Māori. | | | |
|  | Maori have a high prevalence of diabetes and an excess burden of diabetes related morbidity and mortality. Community based lifestyle programmes aimed at reducing this burden are already underway in Christchurch and elsewhere in New Zealand. Knowing what the optimal exercise prescription is for maximising fitness and management of metabolic risk factors, will inform both Maori and non Maori health professionals about the best exercise prescription to incorporate into their lifestyle programmes. | | |
|  | | | |
| F4. Describe the process by which Māori have been engaged in the conception and design of the proposed research. Please identify the group/s with which consultation has taken place and outline their stated view about the proposed research. Please attach their letter/s of support for this specific research project. | | | |
|  | Te Komite Whakarite has been sent a copy of the application for discussion at a meeting of the committee to ensure the study complies with the provisions of the Treaty of Waitangi. In accordance with previous directives from Te Komite Whakarite a clear explanation will be given to Maori participants on collection, storage and disposal of body fluids (blood). In addition, the involvement of whanau during both the consenting process and study visits is understood and this option will be offered to the Maori participants.  Dr Nick Draper and Dr Helen Lunt have consulted xxxxxxxxxx, about study design from the Maori perspective. | | |
|  | | | |
| F4.1 Describe any ongoing involvement the group(s) consulted have in the project. | | | |
|  | | The groups consulted will have no active involvement with data collection during the study, but may be asked to assist with support and guidance for Maori participants. | |
|  | | | |
| F4.2 Describe how information will be disseminated to participants and the group(s) consulted during and at the conclusion of the research project. | | | |
|  | | After initial contact with the participants we will provide a local meeting to discuss the project with individuals/groups. All participants will be offered the option of receiving a report of the finding in the consent form. Te Komiti Whakarite will also be sent a summary of the study findings.  We welcome guidance from xxxxxx and others about the most appropriate methods of disseminating research findings within the Maori community, at the conclusion of the study. | |

#### Responsiveness to ethnic peoples

| F5. What other ethnic groups will be participating in this research based on your sampling frame (for example, Pacific peoples or Asian peoples)? | | | | | | | |
| --- | --- | --- | --- | --- | --- | --- | --- |
|  | All ethnicities, but for reasons discussed above, group activities will be conducted in English. | | | | | | |
|  | | | | | | | |
| F5.1 Are there any aspects of the research based on participation or the | | | |  | Yes | X | No |
| relevance of the research to specific ethnic groups that might raise specific cultural issues? | | | | | | | |
| If **yes**, please outline. If **no**, go to F6. | | |  | | | | |
|  | | | | | | | |
| F5.2 How can this research contribute to reducing inequalities for ethnic peoples in the New Zealand health system? | | | | | | | |
|  | | The exercise prescriptions being studied are low cost and could be undertaken by community leaders with some training (ie by adopting the ‘train the trainers’ concept). Thus the project’s findings could be easily modified for use within specific ethnic communities. | | | | | |
|  | | | | | | | |
| F5.3 Describe what consultation has taken place with specific ethnic group(s) prior to the project’s development and attach evidence of their support. | | | | | | | |
|  | |  | | | | | |
|  | | | | | | | |
| F5.4 Describe any ongoing involvement the group(s) consulted have in the project. | | | | | | | |
|  | |  | | | | | |
|  | | | | | | | |
| F5.5 Describe how you intend to disseminate information to participants and the group(s) consulted at the end of the project. | | | | | | | |
|  | |  | | | | | |

#### Responsiveness to other peoples of interest

| F6. Are there any aspects of the research based on participation or the | | | |  | Yes | X | No |
| --- | --- | --- | --- | --- | --- | --- | --- |
| relevance of the research to specific peoples of interest that might raise specific issues for such communities (for example, for prisoners, people with disabilities, people with diverse sexual identities)? | | | | | | | |
| If **yes**, please outline. If **no**, go to F7. | |  | | | | | |
|  | | | | | | | |
| F6.1 How can this research contribute to reducing inequalities for other peoples of interest in the New Zealand health system? | | | | | | | |
|  |  | | | | | | |
|  | | | | | | | |
| F6.2 Describe what consultation has taken place with specific peoples of interest group(s) prior to the project’s development and attach evidence of their support. | | | | | | | |
|  |  | | | | | | |
|  | | | | | | | |
| F6.3 Describe any ongoing involvement the group(s) consulted have in the project. | | | | | | | |
|  |  | | | | | | |
|  | | | | | | | |
| F6.4 Describe how you intend to disseminate information to participants and the group(s) consulted at the end of the project. | | | | | | | |
|  |  | | | | | | |
|  | | | | | | | |
| F7. Will the study drug/treatment continue to be available to the participant | | | |  | Yes | X | No |
| after the study ends? | | | | | | | |
| F7.1 If **yes**, will there be a cost, and how will this be met? | | |  | | | | |
|  | | | | | | | |
| F7.2 If **no**, why not? | | | Participation in the exercise groups will not be offered outside the study timelines due to resource constraints. We will however endeavour to link interested participants into existing community exercise programmes, as required. | | | | |
|  | | | | | | | |
| F7.3 If there was a placebo arm, what will happen to these participants at the end of the study? | | | No placebo in study design. | | | | |
|  | | | | | | | |
| ***Note: This information needs to be included in the information sheet.*** | | | | | | | |

# Part 3: General

Describe and discuss any ethical issues arising from this project, other than those already dealt with in your answers above.

| Samples will be collected during Phase 1 of the study, to undertake an exploratory analysis of mitochondrial DNA load, in conjunction with the Christchurch Health Laboratories (Dr Richard MacKay, Clinical Biochemist). In brief, subtle variations in mitochondrial DNA content within the general population may partially explain why some people are more fatigued than others, following exercise. It may be possible to increase mitochondrial DNA load with exercise, hence improve fatiguability.  The proposed DNA study measures mitochondrial DNA content in relation to nuclear DNA content, in peripheral blood monocytes. It is therefore not a genetic study in the traditional sense ie it does **not** look at the genetic ‘fingerprint’, unique to that individual. We have however completed Part 6 of the Ethics declaration in good faith, so that the Ethics Committee has information in relation to blood storage, analysis and destruction. |
| --- |

**Thank you for your assistance in helping us assess your project fully.**

Please now complete:

- the declarations (Part 4). If there is more than one site, include a declaration for each site.

If applicable complete:

- a Registered Drug Form
- Form A or B
- Part 5
- Part 6
- Part 7
- Part 8

Attach:

- Checklist to ensure all relevant documents are attached. Incomplete applications will not be reviewed.

# Part 4: Declarations

| Full project title: | Health benefits of high intensity exercise for populations at risk of diabetes and CVD |
| --- | --- |
|  |  |
| Short project title: | Health benefits of high intensity exercise |

## 1. Declaration by principal investigator

| The information supplied in this application is, to the best of my knowledge and belief, accurate. I have considered the ethical issues involved in this research and believe that I have adequately addressed them in this application. I understand that if the protocol for this research changes in any way, I must inform the ethics committee. | |
| --- | --- |
|  |  |
| Name of Principal Investigator (please print): | Dr. Helen Lunt |
|  |  |
| Signature of Principal Investigator: |  |
|  |  |
| Date: | 20.11.09 |

## 2. Declaration by Head of Department in which the Principal Investigator is located or appropriate Dean or other Senior Manager

| I have read the application, and it is appropriate for this research to be conducted in this department. I give my consent for the application to be forwarded to the ethics committee. | | | | |
| --- | --- | --- | --- | --- |
|  | |  | | |
| Name (please print): | | Dr M. Peter Moore | | |
|  |  | |  |  |
| Signature: |  | | Institution: | CDHB |
|  |  | |  |  |
| Date: | 20.11.09 | | Designation: | Clinical Director, Diabetes Services |

- Where the Head of Department is also one of the investigators, the Head of Department declaration must be signed by the appropriate Dean, or other senior manager.
- If the application is for a student project, the supervisor should sign the Head of Department declaration.
- Submit a declaration by the principal investigator for each site.

## 3. Locality organisation approval

| Locality organisation approval is being sought/is attached from the following locations: |
| --- |
| CDHB  University of Canterbury |

# Form A: Declaration of Eligibility of a Clinical Trial for Consideration of Coverage under Accident Compensation Legislation

| **Instructions:** This form is to be completed and the statutory declaration signed by the most senior registered health professional providing or directing the provision of treatment as part of the research. It should be forwarded to the appropriate ethics committee together with the documents seeking ethical approval for the proposed study. The information provided must be sufficiently detailed to enable the ethics committee to be satisfied that the proposed research is not conducted principally for the benefit of the manufacturer or distributor of the medicine or item in respect of which the research is carried out. |
| --- |

The provision of this information will enable the ethics committee to be satisfied that participants in the clinical trial will be considered for coverage under accident compensation legislation for injury caused as a result of their participation in the research.

## Details of proposed research study

| Title of research project: | Health Benefits of high intensity exercise for populations at risk of diabetes and CVD |
| --- | --- |
|  | |
| Name of research director/investigator: | Dr. Helen Lunt |
|  | |
| Location/s of proposed study: | Christchurch Diabetes Centre, 550 Hagley Ave University of Canterbury Dovedale Campus. |
|  | |
| Number of participants: | 104 |
|  | |
| Organisations providing support (in money or kind) for the direct and indirect costs of the research *(please provide names of organisations and details of the type of support provided)*: | HRC District Health Board Research Fund will reimburse a portion of staff time, assay costs and overheads. |
|  | |
| Relationship of proposed research to the pharmaceutical industry or other company involved in health research *(please describe the involvement of industry in your proposed research and provide details of support to be received from them)*: | No relationships |

#### Statutory declaration

I Dr Helen Lunt of Christchurch, New Zealand solemnly and sincerely declare that as the most senior registered health professional providing or directing the provision of treatment as part of the research, the proposed study is not conducted principally for the benefit of the manufacturer or distributor of the medicine or item in respect of which the trial is carried out. I make this solemn declaration conscientiously believing the same to be true and by virtue of the Oaths and Declarations Act 1957.

| Helen Lunt |  |  |  |  |
| --- | --- | --- | --- | --- |
| Name *(please print)* |  | Signature |  | this day of |

before me

|  |  |  | |
| --- | --- | --- | --- |
| Name of witness *(please print)* |  | Signature of witness | |
|  | |  | |
| a Justice of the Peace, or | |  |  |
|  | |  | |
| a Solicitor of the High Court | |  |  |
|  | |  | |
| or other person authorised to take a statutory declaration. | |  |  |

**Warning:** Please note that it is an offence under part VI subsection 111 of the Crimes Act 1961 to make a false statutory declaration. **Note:** Applicants conducting a research study that is conducted principally for the benefit of the manufacturer or distributor of the medicine or item in respect of which the trial is carried out should complete Form B.

# Form B: Declaration of Provision of Compensation for Injury for Participants in a Research Study for a Pharmaceutical Company or any Other Company Involved in Health Research

| **Instructions:** This form is to be completed and the statutory declaration signed by the applicant. It should be forwarded to the appropriate ethics committee together with the documents seeking ethical approval for the proposed study and appropriate assurance from the pharmaceutical company or any other company involved in health research. The information provided must be sufficiently detailed to enable the ethics committee to be satisfied that:   - the proposed research is conducted principally for the benefit of the manufacturer or distributor of the medicine or item in respect of which the research is carried out - participants in the proposed research project will receive an acceptable level of compensation from a pharmaceutical company or any other company involved in health research in the event of injury to participants resulting from their involvement in the proposed research project. - researchers and institutions have indemnity cover to provide an acceptable level of compensation in the event of injury to participants resulting from any researcher or research staff deviating substantially from the trial protocol. |
| --- |

## Details of proposed research project

| Title of research project: |  |
| --- | --- |
|  | |
| Name of research director/investigator: |  |
|  | |
| Location of proposed study: |  |
|  | |
| Number of participants: |  |
|  | |
| Organisations providing support (in money or kind) for the direct and indirect costs of the research *(please provide names of organisations and details of the type of support provided)*: |  |
|  | |
| Relationship of proposed research to the pharmaceutical industry or other company involved in health research *(please describe the involvement of industry in your proposed research and provide details of support to be received from them)*: |  |
|  | |
| Details of compensation to be provided to participants in the event of injury *(documents signed by the sponsoring pharmaceutical company or other company involved in health research must be attached)*: |  |

#### Statutory declaration

I       (name) of       (town/city) solemnly and sincerely declare that as director of the proposed research, the proposed study is conducted principally for the benefit of the manufacturer or distributor of the medicine or item in respect of which the trial is carried out and that in the event of injury arising from their participation in the research, an appropriate level of compensation, in line with the *New Zealand Researched Medicines Industry Guidelines on Clinical Trials – Compensation for Injury Resulting from Participation in Industry Sponsored Clinical Trials,* will be provided by       (name of pharmaceutical company or another company involved in the research project) as detailed in the attached documents, unless the injury is a result of a significant deviation from the study protocol. I confirm that I, my research staff and the host institution have indemnity insurance that covers injury as a result of significant deviation from the study protocol. I make this solemn declaration conscientiously believing the same to be true and by virtue of the Oaths and Declarations Act 1957.

|  |  |  |  |  |
| --- | --- | --- | --- | --- |
| Name *(please print)* |  | Signature |  | this day of |

before me

|  |  |  | |
| --- | --- | --- | --- |
| Name of witness *(please print)* |  | Signature of witness | |
|  | |  | |
| a Justice of the Peace, or | |  |  |
|  | |  | |
| a Solicitor of the High Court | |  |  |
|  | |  | |
| or other person authorised to take a statutory declaration. | |  |  |

**Warning:** Please note that it is an offence under part VI subsection 111 of the Crimes Act 1961 to make a false statutory declaration. **Note:** Applicants conducting a research study that is not conducted principally for the benefit of the manufacturer or distributor of the medicine or item in respect of which the trial is carried out should complete Form A.

# Form for Registered and Unregistered Medicines *(Refer Question B19)*

## Information required for trials involving administration of medicines currently registered in New Zealand

**This form is be completed for all medicines including non-registered medicines except where the medicine will be given regardless of entry into the trial (eg, anaesthetic) and that medicine is not being studied in any way**

| Trade name of medicines |  |
| --- | --- |
|  | |
| Generic name of medicines |  |
|  | |
| Pharmacological class |  |
|  | |
| Form of administration used in the study |  |
|  | |
| Recommended dose range |  |
|  | |
| Contraindications |  |
|  | |
| Known or possible interactions with non-trial medicines the participants may be taking |  |
|  | |
| Common side effects and serious adverse reactions |  |
|  | |
| Additional information, eg, long half-life, immunosuppression |  |
|  | |

**Appendix 1.**

***Research Study Design continued (from section A.3.1)***

**Phase 1 – details of exercise intervention**

The exercise interventions will be based on previously successful protocols, undertaking in the exercise physiology laboratory. In phase one, 60 participants will be randomly assigned to LIET (low intensity exercise) (N = 20), MVIT (maximal volitional intensive training) (N=20) or high intensity training (N = 20). All subjects will undergo a standardised, supervised paired maximal intensity exercise treadmill test to measure VO2max and paired blood tests, before and immediately after the 12 week intervention. The exercise intervention will be undertaken in groups of around 6 participants per group.

The three exercise prescriptions will be isocaloric ie the same overall energy expenditure will take place per session in all three groups but the method, hence time requirements to achieve this will vary. All groups will complete 3 supervised training sessions per week, based in Hagley Park. Exercise groups will be instructed by health and physical activity undergraduates from CPIT, Canterbury or Lincoln University who will all have current First Aid qualifications and will be given training and guidance on how to organise and conduct the training sessions. All participants will exercise with the aid of a heart rate monitor, thereby allowing the participants to control their corresponding exercise heart rate relative to VO2peak. The instructors will monitor each participant’s heart rate during exercise to ensure training sessions are carried out in the desired heart rate throughout the 12 week training period. As fitness improves, the exercise load ie intensity of exercise will be increased.

**High intensity exercise groups (HIITT and MVIT).** The HIIT and MVIT groups will complete a warm-up for 5 min (at an intensity of 50-60% VO2peak or 65-75% heart rate peak). The HIIT group will then walk for four intervals of 4 min at 80-90% VO2peak (85-95% heart rate peak), interspersed with 3 recovery periods of 3 min at 50-60% VO2peak. The MVIT group will exercise ‘as hard as they can’ ie at a similar intensity to the final minute of the exercise treadmill test undertaken at baseline, for 60 second intervals, followed by a cool down period then further maximal intensity exercise over the course of the session. At the end of the session, HIIT and MVIT subjects will complete a cool-down of 3 min at 50-60% VO2peak.

**Low intensity exercise group.** The LIET group will complete walking at an intensity of 50-60% for the time it will take to burn off similar calories to the HIIT and MVIT groups. (The approximate value will be ~41 min which follows previous research paradigms (Rognmo *et al*, 2004) and is based on the average VO2peak for all groups measured at baseline).

**Phase 2**

In phase two, results from phase one will be used to fine-tune the testing and training protocols. In this phase 44 new to the study participants will be randomly assigned into 2 groups; the best high intensity programme from Phase (ie HIIT or MVIT) and a control group undertaking LIET. There will be 22 participants in each group. Subjects will undertake blood testing and VO2 max as for Phase one at times 0, 13 and 26 weeks. The second VO2max test done at 13 weeks will be used to check progress and adjust the training intensities for the final 3 months of training. Once a week each exercise group will come together at a local venue (Halswell Health Medical Centre or the Whanau Tautoko Charitable Trust) where instructors (trained undergraduates from CPIT, Canterbury or Lincoln University who will all have current First Aid qualifications) will take the group through their training for that day. The other two exercise sessions each week will take place at a venue chosen by the individual participants, either at the participant’s home or in a convenient community location. All participants will exercise with the aid of a heart rate monitor thereby allowing the participants to control their corresponding exercise heart rate relative to VO2peak. The instructors will monitor each participant’s heart rate each week to ensure training sessions are carried out in the desired heart rate throughout the 6 month training period

See next page for the testing protocols.

**Study outline – Phase 1**

**Week**

0

2

14

12 week walking based exercise programme

**Key:**

Randomisation and group allocation, timetable details finalised for exercise groups

Fasting venesection, anthropomorphic measurements

and questionnaires

Exercise treadmill testing - measurement of VO2 max

**Initial screening and consent**

Potential subjects will be pre-screened by phone to assess their study eligibility ie to determine whether or not they fulfil study inclusion criteria. This pre screening will be undertaken by the research nurse or one of the named investigators. Potential subjects will then be invited to a group meeting at the Christchurch Diabetes Centre to discuss the study, including the need for exercise testing. Potential participants will be encouraged to ask questions about the study. They will be given the information and consent sheets at that meeting. Potential participants will be encouraged to discuss the study with their GP. Potential participants can then either complete the consent sheet at that meeting or, if they prefer, send it back in an s.a.e., to be countersigned by the investigator at the first baseline testing visit (see below). It is anticipated that up to 10 such group meetings will be required to enable screening of sufficient numbers of potential participants.

**Baseline Testing**

About a week prior to baseline testing, consenting subjects will be sent a four day food record and instructions about completing this, the quality of life and health and exercise history questionnaires and the facilitators and barriers to change questionnaire. They will also be given instructions about undertaking an overnight fast. Subjects will bring their completed questionnaires to the first study visit.

Day -1. Arrive at Diabetes Clinic (550 Hagley Ave) from 7am-9am in fasted state.

Hand in completed records and questionnaires, which will be checked for completeness by the researchers. Any outstanding issues with the consent forms will be completed prior to venesection. A

fasting blood sample will be taken and anthropomorphic measurements will also be done in the fasting state. Subjects will be offered a light breakfast, as required and any outstanding paperwork will then be completed.

It is anticipated that up to 10 subjects will be screened on any given morning.

Day 0. Arrive at Canterbury University (College of Education Exercise laboratory, Dovedale Ave) between 7am-10am either fasting or after a light breakfast (subject’s preference). Complete Maximal Treadmill Test.

Facilities and staff are available to undertake testing in up to twelve subjects per morning.

Days 2-14. Rest – await results of randomisation and group allocation.

Days 2-14 Start the exercise programme within a fortnight of randomisation.

**Exit interview for early termination of the study**

Subjects who elect to finish the programme early will be asked to undertake a (voluntary) exit interview, to better understand their likes and dislikes of the programme.

**Post Testing (after 12 weeks)**

Follow the same schedule as above for the baseline testing ie fasting venesection etc at Diabetes Centre, followed the next day by measurement of VO2 max.

**Variables to be Measured**

*Questionnaires.* The following validated questionnaires will be used:

*Fasting venous blood collection*

1. Insulin

2. Fasting glucose

3. Insulin sensitivity (HOMA %)

4. B-cell function (HOMA %)

5. Blood lipid profile (HDL, LDL, VLDL, triglycerides)

6. Highly sensitive C-reactive protein

7. DNA collection, for storage for mitochondrial depletion studies

*Anthropometric Measurements*

1. Height, weight, waist and hip circumferences.

2. Bio-impedance measurement (Fat free mass, fat mass, % body fat)

*Physiological Measurements before, during and after VO2max Test*

1. Resting: HR, BP, SV, Q (Finapres Finometer)

2. During Exercise: HR, VO2, RER, VE Perceived exertion.

3. Post-exercise: Perceived exertion, HR

*Physiological Measurements before, during and after Submax Test*

1. Resting: HR, BP, SV, Q (Finapres Finometer)

2. During Exercise: HR, BP, SV, Q, VO2, RER, VE

3. Post-exercise: HR, BP, SV, Q, VO2, RER, VE

From the VO2max test we will calculate the VO2peak, time to exhaustion, HRpeak while from the submaximal test we will calculate workload efficiency and cardiovascular stress at 50% of VO2peak.

**Phase 2.**

The subjects will be randomly selected to one of 2 groups (Low intensity control group and High-intensity intervention group). The exercise prescription for these two groups will be based on the outcomes from phase 1 of the study and will be for 26 weeks rather than twelve weeks. The measurement of V02 max, fasting blood samples, questionnaires and anthropomorphic measurements will be as for Phase 1, but undertaken at 0, 13 and 26 weeks. Mitochondrial depletion studies will not be undertaken for Phase 2 (ie they will be done for Phase 1 only). The only location that will change is the location for the exercise prescription, which will now be near the subjects’ own home ie around the Aranui or Halswell area.
