# Supplementary material for: High Intensity Interval Training in a Real World Setting: A Randomized Controlled Feasibility Study in Overweight Inactive Adults, Measuring Change in Maximal Oxygen Uptake
Source: PLoS One. 2014 Jan 13;9(1):e83256. doi: 10.1371/journal.pone.0083256 (PMC3890270; doi:10.1371/journal.pone.0083256)
Supplement: Table S1 — Number (description) of participants experiencing an adverse event by exercise group allocation. aEvent of sufficient severity that participant exited study early. bEvent of sufficient severity that participant did not undertake exit O2max. (DOCX) [file pone.0083256.s003.docx]

|  | **Low intensity walking (WALK)** | **Aerobic interval training (AIT)** | **Maximum volitional intensity training (MVIT)** |
| --- | --- | --- | --- |
| Related to intervention | 1 (*Shin splints*) | 3 (*Ankle sprain x2*) (*Calf strain*) | 4 (*Iliotibial band syndrome*) (*Achilles tendonitis* ^a,b^) (*Bilateral flexor tendinitis*) (*Plantar fasciitis* ^a,b^ ) |
| Not directly related to exercise intervention | 1 (*Fractured coccyx* ^a,b^ *)* | 0 | 2 (*Bronchitis* ^a,b^ ) (*Bronchopneumonia* ^b^) |
