# Supplementary material for: High Intensity Interval Training in a Real World Setting: A Randomized Controlled Feasibility Study in Overweight Inactive Adults, Measuring Change in Maximal Oxygen Uptake
Source: PLoS One. 2014 Jan 13;9(1):e83256. doi: 10.1371/journal.pone.0083256 (PMC3890270; doi:10.1371/journal.pone.0083256)
Supplement: Text S1 — Ancillary methodological details. (DOCX) [file pone.0083256.s004.docx]

***Clinical and anthropometric measurements and questionnaires***

A brief clinical history, including current medications, was documented by the physician (HL) at a pre-study clinic screening visit. Participants attended the research clinic fasting but were instructed to take their usual morning medications. Sitting blood pressure ((HEM-907, Omron, Matsuzaka City, Japan), waist and hip circumference, height and weight were measured and BMI calculated. Percentage body fat was measured using a bioimpedance method (InBody 230, Biospace, Seoul, Korea). Fasting blood samples were taken and either analyzed immediately for hemoglobin and hematocrit, or placed on an ice/water slurry followed by refrigerated centrifugation within 10 minutes then frozen at minus 20^o^C for batched analysis of lipids, glucose and insulin at an accredited laboratory. Hemoglobin and hematocrit were measured to ensure no participants were anemic and to record change in these variables during the study period, as major change might impact on interpretation of changes in O_2max_. Quality of life was measured using the SF36 (SF-36v2, qualitymetric.com). The PAR-Q & You (Physical Activity Readiness questionnaire, [www.csep.ca/english/view.asp?x=698](http://www.csep.ca/english/view.asp?x=698)) was used as a measure of disease burden.

***Adherence with the exercise prescription***

When participants attended group sessions, exercise duration (minutes) was recorded by the exercise group leaders. The heart rate response to exercise using downloadable HR monitors was used as the measure of exercise intensity. The exercise group leaders also kept a log of participants’ attendance at group sessions, including group ‘catch up’ sessions and also logged individual’s unsupervised, non-group based exercise sessions. Downloadable HR monitors were used to measure the proportion of time spent within the allocated HR zones for the WALK (walking) and AIT (aerobic interval training) groups and as an indirect measure of exercise intensity for the MVIT (maximal volitional intensity training) group. Participants replaced their standard HR monitor with a downloadable S610 monitor for one exercise session during weeks 6,9,11 i.e. for three sessions over the duration of the study. Participants and exercise group leaders were blind to participants’ HR monitor results for these three sessions.

***Adherence with the request not to undertake lifestyle change outside intervention sessions***

Participants were requested not to change their food quality or quantity and not to alter their usual physical activities. Change in food intake, including total daily energy intake, was assessed by comparing results from a four day food diary before and upon completion of the study. The dietitian reviewed diaries with participants at the exit assessment then undertook nutritional analysis using nutritional software (FoodWorks® Professional 2009, www.xyris.com.au). Pedometers were used to determine whether there were any group differences in incidental activity occurring outside the prescribed exercise sessions that might confound interpretation of group differences in O_2max_. Participants recorded pedometer data over a one week period at week 5-6 and again at week 11-12. Participants were instructed to wear their pedometers at all times over the 7 days of pedometer data collection, except for the led exercise sessions and any water based activities.

***Monitoring of Adverse Events / Harms***

There are no agreed criteria for the reporting of harms (adverse events) in exercise studies.^1,2^ Data on minor, transient harm may be difficult to capture as it may include self-limiting discomfit due to introduction of the exercise program as well as early manifestations of exercise injury. A pragmatic decision was therefore made to record all injuries potentially related to the study interventions. Participants completed a questionnaire at week 2 or 3 of the study, focusing on physical symptoms. Exercise group leaders kept a diary of reasons for non-attendance at group sessions, including possible injuries and they reported harms to the supervising exercise physiologist. Persistent or significant symptoms were reported to the physician for further evaluation, including possible relatedness to the individual’s exercise prescription. Participants were reminded not to discuss their group allocation with the physician. The physician also reviewed possible additional harm and injury at each participant’s exit assessment. The section of the SF36 reporting body pain was used as a further measure of harm.

***Blinding of clinical assessors to details of the exercise prescription***

The Participant Information Sheet excluded details of exercise load and number of repetitions inherent in the three exercise prescriptions, WALK, AIT and MVIT. Participants received these details from the exercise physiologists at their first supervised exercise session but the clinical assessors (physician, nurses, dietitian) did not have access to these details until database lock had been achieved, thus ensuring blinding of these assessors.

***References***

1. Janney CA, Jakicic JM. (2011) The influence of exercise and BMI on injuries and illnesses in overweight and obese individuals: a randomized control trial. Int J Behav Nutr Phys Act. Doi: 10.1186/1479-5868-7-1.
2. Goodrich DE, Larkin AR, Lowery J, Holleman RG, Richardson CR*.* (2007) Adverse events among high-risk participants in a home-based walking study: a descriptive study. Int J Behav Nutr Phys Act.  Doi: [10.1186/1479-5868-4-20](http://dx.doi.org/10.1186%2F1479-5868-4-20)
